# The modified Borg cycle strength test (mBCST): Feasibility and physiological response in people with COPD and healthy older adults

**DOI:** 10.1113/EP092151

**Published:** 2025-07-10

**Authors:** Jana De Brandt, Johan Jakobsson, Mattias Hedlund, Thomas Sandström, André Nyberg

**Affiliations:** ^1^ Department of Community Medicine and Rehabilitation, Faculty of Medicine Umeå University Umeå Sweden; ^2^ Lung and Allergy Clinic Norrlands Universitetssjukhus Umeå Sweden; ^3^ Department of Public Health and Clinical Medicine Faculty of Medicine, Umeå University Umeå Sweden

**Keywords:** chronic obstructive pulmonary disease, exercise prescription, exercise test, high‐intensity interval training

## Abstract

Accurate prescription of supramaximal exercise requires exercise tests covering the intensity domain between maximal aerobic and peak power output. All‐out tests are commonly used for this objective but are considered challenging for people with chronic obstructive pulmonary disease (COPD) due to the extreme physiological demand. The modified Borg cycle strength test (mBCST), previously used in older adults to achieve supramaximal intensities, might be a suitable alternative in people with COPD. We aimed to determine the feasibility of the mBCST in people with COPD and to compare the physiological response with that of healthy older adults. Eighteen people with COPD and 16 age‐, sex‐ and physical activity‐matched healthy adults performed a cardiopulmonary exercise test and a mBCST. The mBCST is an incremental test [30 s:30 s cycling:rest; with individualized starting load and step size (15–50 W)] with end‐of‐test criteria of a rating of perceived exertion of ≥17 or cadence of <75 RPM for >5 s. Feasibility was assessed using a framework covering the aim, interpretability, familiarity, duration, scoring/completion complexity, costs and safety. Measurements of external exercise intensity, rating of perceived exertion, symptoms and cardiorespiratory demand were obtained. The mBCST was deemed feasible according to the feasibility framework. Expressed relative to the cardiopulmonary exercise test, the participants reached supramaximal external exercise intensities during the mBCST [COPD, 145 (125–168)%; healthy, 154 (148–163)%], without differences in intensity or physiological response between groups (*p *> 0.05). The mBCST is feasible in people with COPD and enables supramaximal external exercise intensities, with similar physiological response to healthy older adults. The mBCST could be considered when selecting an exercise test to prescribe supramaximal exercise.

## Abstract (highlights)

- **What is the central question of this study?**
  All‐out tests are recommended for prescribing supramaximal exercise but are considered challenging for people with chronic obstructive pulmonary disease (COPD), necessitating alternatives. Is the modified Borg cycle strength test (mBCST) feasible and applicable for prescribing supramaximal exercise in people with COPD, and is the physiological response to the mBCST different between people with COPD and healthy older adults?
- **What is the main finding and its importance?**
  In people with COPD, the mBCST demonstrated high feasibility and enabled supramaximal exercise >150% of maximal aerobic power with a similar physiological response to that of healthy older adults. This highlights its applicability for prescribing supramaximal exercise and could be considered when selecting an appropriate test.

## 1 INTRODUCTION

Endurance training is a key component of pulmonary rehabilitation in people with chronic obstructive pulmonary disease (COPD) (Gloeckl et al., 2023; Rochester et al., 2023; Spruit et al., 2013), aiming to improve aerobic exercise capacity for daily life activities while also enhancing peripheral muscle function, reducing symptoms and improving quality of life (De Brandt et al., 2018; McCarthy et al., 2015; Rochester et al., 2023; Spruit et al., 2013; Vaes et al., 2019, 2011).

Traditionally, moderate‐intensity continuous or interval training has been prescribed for people with COPD (Gloeckl et al., 2023; Spruit et al., 2013). Independent of the selected endurance training modality, a key goal is to reach sufficiently high exercise intensities to enable physiological adaptations (Casaburi & ZuWallack, 2009; Morris et al., 2016). However, this is particularly challenging for people with COPD (Sawyer et al., 2020). Several factors contribute to this difficulty, but ventilatory limitations and altered respiratory mechanics during exercise are key contributory factors (O'Donnell et al., 2016) that limit the achievable exercise intensity and often lead to suboptimal cardiovascular and muscular stress needed for physiological adaptations (exercise principle of overload) (American College of Sports Medicine, 2022). As a result, alternative endurance training strategies [e.g., high‐intensity interval training (HIIT), downhill walking, partitioning, addition of non‐invasive ventilation] have been developed (Gloeckl & Osadnik, 2021) to maximize endurance training outcomes for people with COPD.

Recently, a novel concept known as SupraHIIT has been introduced, entailing short‐duration HIIT. SupraHIIT differs from traditional HIIT and moderate‐intensity continuous training (MICT) by performing exercise training at intensities exceeding an individual's maximal aerobic power (MAP). The MAP is defined as the achieved power output that elicits maximal oxygen uptake during a cardiopulmonary exercise test (CPET) (Sandford et al., 2021). SupraHIIT has been shown to deliver comparable or superior health benefits to MICT in healthy adults (Frykholm et al., 2024; MacInnis & Gibala, 2017; Simonsson et al., 2023), and it has also recently been shown to be feasible among people with COPD, enabling exercise intensities more than three times higher compared with MICT (Jakobsson et al., 2025). However, given that the exercise intensity in the supra‐MAP domain uses anaerobic power reserves (Sandford et al., 2021), prescribing SupraHIIT should be based on exercise tests conducted within this specific intensity domain, delimited by MAP as the lower limit and peak power output as the upper limit (Sandford et al., 2021).

Exercise tests within the anaerobic power reserve often consist of an all‐out test, such as the Wingate anaerobic test. All‐out tests, however, are considered challenging for older adults and people with COPD due to their extreme physiological demands, high and disabling end‐test dyspnoea scores, high and uncontrolled pedalling cadence, and substantial motivation requirements (Bayati et al., 2011; Chura et al., 2012; Hirsch et al., 2021). The modified Borg cycle strength test (mBCST) is a less strenuous alternative to all‐out tests, using a submaximal, stepwise incremental approach with controlled cadence to estimate maximum mean power output over 30 s (MPO30) (Borg, 1982), with later refinements showing its applicability for prescribing SupraHIIT in healthy older adults (Frykholm et al., 2024; Hedlund et al., 2019; Simonsson et al., 2023).

Taken together, although the mBCST seems feasible and effective in prescribing SupraHIIT in healthy older adults, its applicability in people with COPD remains to be determined. Therefore, we aimed to determine the feasibility of the mBCST in people with COPD and its applicability in enabling supramaximal external exercise intensities. The latter is achieved by comparing the external exercise intensity achieved during the mBCST with the MAP achieved during a traditional CPET. Secondly, we aimed to determine and compare the physiological response between the mBCST and CPET, and between people with COPD and age‐, sex‐ and physical activity‐matched healthy older adults.

## 2 MATERIALS AND METHODS

### 2.1 Study design

We conducted a cross‐sectional study using within‐ and between‐group comparisons. The sample included participants with COPD and age‐, sex‐ and physical activity‐matched healthy older adults. Data collection took place between March and December 2022, and the study is reported in accordance with the STROBE guidelines (von Elm et al., 2007). This study is part of a cross‐over trial (ClinicalTrials.gov Identifier: NCT05874999) (Jakobsson et al., 2025), and some findings have previously been disseminated in the form of conference proceedings (De Brandt et al., 2023). Ethical approval was obtained from the Swedish Ethical Review Authority (reference: 2021‐05408‐02), and the study was conducted in accordance with the principles outlined in the *Declaration of Helsinki*. Written informed consent was obtained from all participants prior to study enrolment.

### 2.2 Participants

Study participants were selected using a convenience sampling method (for more details, see Supporting Information). Participant eligibility was based on the following inclusion criteria: (1) ≥60 years of age; and (2) diagnosis of COPD based on post‐bronchodilator ratio of forced expiratory volume in 1 s over forced vital capacity (FEV_1_/FVC) < 0.7 (GOLD, 2024) (additional COPD‐specific inclusion criteria). Exclusion criteria were as follows: (1) neuromuscular, orthopaedic and/or any other condition that compromises participation in exercise testing; (2) unstable cardiac disease and/or the presence of a cardiac stimulator; and (3) diagnosis and treatment of lung cancer in the last 5 years. Additional group‐specific exclusion criteria were as follows: an acute exacerbation of COPD in the last 6 weeks (COPD); and presence of any respiratory disease or too low or high number of steps per day that prohibited physical activity matching (healthy older adults). Details on the assessment of physical activity can be found in the Supporting Information.

### 2.3 Procedure

Data collection was conducted during a single visit. First, lung function testing and CPET were performed at the Unit of Clinical Physiology at the University Hospital of Umeå (Umeå, Sweden). After a 90 min break, assessment of participant characteristics, including body composition, and a second cycling test (i.e., mBCST) were performed at the Umeå Movement and EXercise laboratory at Umeå University (Umeå, Sweden). The procedure for the mBCST is detailed below. For information regarding the other assessments, please refer to the Supporting Information.

#### 2.3.1 mBCST

**FIGURE 1:**
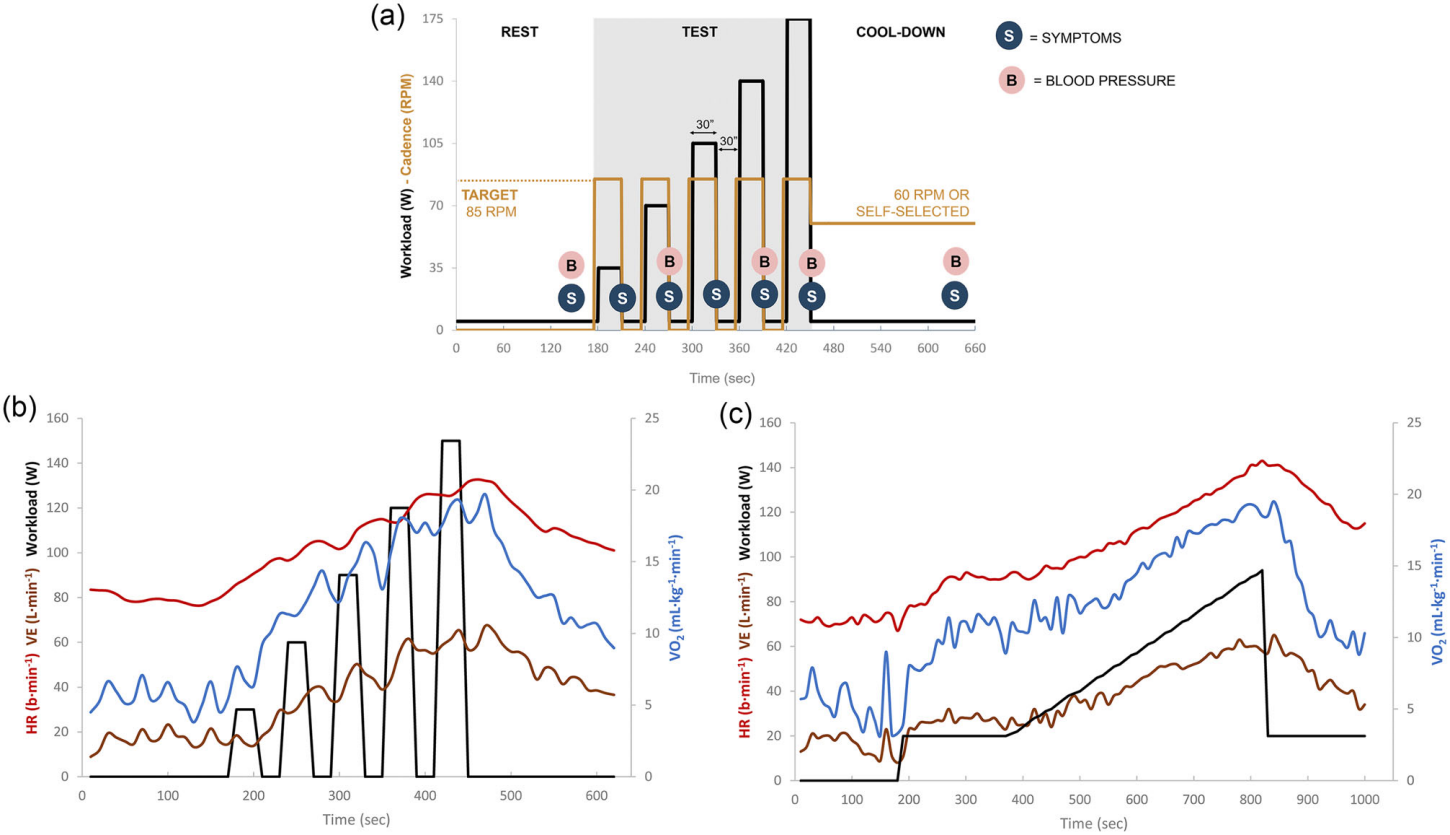
(a) Illustrative visualization of a mBCST protocol, starting at 35 W and with step increments of 35 W, achieving five steps, accompanied by timings of obtainment of symptom ratings and blood pressure. (b, c) Graphical representation of a mBCST protocol starting at 30 W and with step increments of 30 W, achieving five steps; and a CPET protocol, starting at 20 W and with a ramp increment of 10 W/min, in a person with COPD. Abbreviations: COPD, chronic obstructive pulmonary disease; CPET, cardiopulmonary exercise test; HR, heart rate; mBCST, modified Borg cycle strength test; RPM, revolutions per minute; ${\dot{V}}_{E}$, ventilation; ${\dot{V}}_{O_{2}}$, rate of oxygen consumption.

The mBCST is a submaximal, stepwise incremental test for estimation of maximum MPO30 without requiring maximal effort (Figure 1; template in Supporting Information). The mBCST was performed on a stationary mechanically braked ergometer (Monark LC6, Vansbro, Sweden). Starting loads and incremental steps (maximum of 10 steps) ranging from 15 to 50 W were used and chosen based on the MAP of each participant obtained during the CPET, with the goal to reach at least three steps (see template mBCST and mBCST workload step increment calculator in Supporting Information). Participants wore a facemask (Vyaire, Mettawa, IL, USA) for breath‐by‐breath gas analysis via indirect calorimetry (Metamax‐3B, Cortex Biophysik, Leipzig, Germany). Additional monitoring included a heart rate (HR) sensor (H9, Polar Electro Oy, Kempele, Finland), an automatic blood pressure cuff (Tango M2, Suntech Medical, Morrisville, NC, USA) and a finger sensor for peripheral oxygen saturation (Wristox 3150, Nonin, Plymouth, MN, USA). Gas calibration and automatic volume calibration of the metabolic cart were performed before every test.

First, participants completed a familiarization bout at the first incremental step, where they learned to ramp up to the target pedalling cadence of 80–90 RPM. Next, a forced spirometry was performed for operational lung volume assessment during exercise (Guenette et al., 2013). Then, after a 3 min resting phase, the test commenced with 30 s intervals of incremental workload steps at a pedalling cadence of 80–90 RPM, alternating with 30 s of passive rest. The test continued until one of the following stop‐criteria was met: rating of perceived exertion (RPE) ≥ 17 points or pedalling cadence < 75 RPM for >5 s (the reason for slowing down was asked, if the latter was the case). The test was concluded by 3 min of unloaded cool‐down at a slow, self‐selected pedalling cadence.

Blood pressure, RPE, rating of dyspnoea and leg fatigue (Borg CR10), in addition to inspiratory capacity (IC) manoeuvres were obtained throughout the test (see details in Supporting Information). Standardized encouragement was given during the test.

#### 2.3.2 Outcome variables: Data processing and presentation

Outcome variables at isoMAP (mBCST data point closest to MAP; mBCST only) and at the end of exercise, including time to exhaustion (in seconds), workload (in watts), rate of oxygen consumption (${\dot{V}}_{O_{2}}$; in millilitres per kilogram per minute), HR (in beats per minute), oxygen pulse (in millilitres per beat), systolic blood pressure (in millimetres of mercury), ${\dot{V}}_{E}$ [in litres per minute; as a percentage of maximal ventilatory ventilation (MVV)], respiratory rate (in breaths per minute), Δ_end‐rest_ IC (in litres), peripheral oxygen saturation (as a percentage), ventilatory equivalents [V˙E/V˙EV˙O2V˙O2, V˙E/V˙EV˙CO2V˙CO2 and V˙E/V˙EV˙CO2V˙CO2 nadir (CPET only)], respiratory exchange ratio (RER), RPE (6–20 points) and Borg CR10 dyspnoea and leg fatigue (0–10 points) were collected during the CPET and mBCST. The data processing is provided in Table S1. Values are reported as absolute, percentage of predicted (CPET) and percentage of CPET (mBCST).

The percentage of predicted values were calculated using equations for ${\dot{V}}_{O_{2}}$ (Gläser et al., 2013), MAP (Brudin et al., 2014) and HR (Tanaka et al., 2001). Maximal effort during CPET was confirmed by meeting at least one European Respiratory (ERS)/American Thoracic Society (ATS) criterion (Radtke et al., 2019). An abnormal exercise response was defined as peak ${\dot{V}}_{O_{2}}$ or MAP < 85% predicted, followed by a determination of exercise limitation.

#### 2.3.3 Feasibility of mBCST

Feasibility was assessed by participants reaching a supramaximal intensity (>100% MAP) and using the test quality framework of Robertson et al. (2017), including the following feasibility criteria: interpretability, familiarity required, duration, scoring complexity, completion complexity and cost. Adverse events were reported as a safety criterion. Table 1 details feasibility criteria and measurements.

**TABLE 1:** Feasibility of the modified Borg cycle strength test.

Feasibility criteria	Definition	mBCST	Results	
			COPD (*n* = 18)	Healthy older adults (*n* = 16)
Reaching supramaximal intensity	The achievement of a mBCST workload > MAP	Achieved mBCST workload in watts^a^	175 ± 63 W	256 ± 55 W
		Percentage of participants that achieve a mBCST workload > MAP^a^	100%	100%
Interpretability	The degree to which practical meaning can be assigned to a test result	Description of practical meaning of mBCST‐achieved workload	mBCST‐achieved workload: This is the power output somebody could maintain for 30 s corresponding to an RPE = 17. Within our study, the determination of the mBCST‐achieved workload is needed for the estimation of maximum MPO6 using the formula: mBCST‐achieved workload × 1.75, to be able to subscribe SupraHIIT according to Simonsson et al. (2023)	
Familiarity required	The need to undertake a test familiarization session with all participants prior to main testing in order to reduce or eliminate learning or reactivity effects	Percentage of participants that completed the familiarization bout	94%	100%
		Reasons for failing the familiarization	Not able to reach target RPM (*n* = 1)	NA
		Description of familiarization with RPE scale	All participants were already familiarized with the RPE scale, because the same scale was explained before, and used during CPET. Additionally, the RPE scale was explained again to the participants before the start of the mBCST.	
Duration	Expected and/or real duration of the testing protocol	Absolute test time (without rest and recovery), in seconds^a^	274 ± 32 s	324 ± 44 s
		Total test time (with rest and recovery), in seconds^a^	634 ± 32 s	684 ± 44 s
Scoring complexity	The ease with which a test can be conducted and scored in a practical setting by the test administrator	Percentage of test conduction problems	12%	0%
		Reasons for test conduction problems	Encouragement of test administrator was misinterpreted by participant, leading to a pause in pedalling during an interval (n = 1) Technical problem with bike (n = 1)	NA
		Percentage of participants with RPE stop‐criterion^a^. When this stop‐ criterion is used, the scoring complexity is low because the achieved mBCST workload can be used directly to calculate the estimated maximum MPO6	69%	44%
		Percentage of participants with RPM stop‐criterion^a^. When this stop‐ criterion is used, the scoring complexity is higher because an extra calculation step is needed to provide the achieved mBCST workload, which is used to calculate the estimated maximum MPO6	31%	56%
Completion complexity	The ease with which a test can be completed by a participant	Percentage of participants that complete the test^b^	94%	100%
		Reason for non‐completion of test/non‐valid test^b^	Not able to reach at least 3 steps (n = 1) [Correction added on 17th October 2025 after first online publication: The value of *n* has been updated from 3 to 1.]	NA
Cost	The total amount of resources required for test administration including equipment, time and administrator expertise/experience	Description of needed equipment, monitoring equipment and expertise of test administrator	Within our study we used the following: Equipment: a mechanically braked ergometer that is able to ramp up to a maximal load of 500 W within 2 s and RPE 6–20 scale Monitoring equipment: Heart rate sensor, automatic blood pressure device with ECG trigger, finger pulse oximeter, BORG CR 10 dyspnoea and leg fatigue scale Test administrator's expertise: Master degree in Sport Sciences or alike Advanced expertise in exercise testing Novice expertise in mBCST testing	
Safety	The rate of adverse events during the test protocol	Percentage of adverse events	6%	6%
		Adverse events	Saddle pain (*n* = 1)	High heart rate during cool‐down (n = 1)
		Category of adverse events: (1) mild and temporary; (2) serious symptoms and potential risk of severe injury or life‐threatening; (3) manifest injury or disease; (4) death (Littbrand et al., 2006)	Mild	Mild

*Note*: Data are expressed as the mean ± SD or percentages.

Abbreviations: COPD, chronic obstructive pulmonary disease; ECG, electrocardiography; HIIT, high‐intensity interval training; mBCST, modified Borg cycle strength test; MPO30, mean power output over 30 s; MPO6, mean power output over 6 s; NA, not applicable; RPE, rating of perceived exertion; RPM, revolutions per minute.

^a^Non‐completers and persons who were not able to perform the familiarization session are excluded.

^b^Persons who were not able to perform the familiarization session are excluded.

### 2.4 Statistical methods

The Statistical Package for the Social Sciences software (IBM, Chicago, IL, USA) was used to perform statistical analyses. Numerical data are described as the mean (SD) or median (quartile 1–quartile 3). Categorical data are described as number (percentage).

Categorical data between groups were compared using the χ^2^ test for homogeneity (3 × 2 comparisons) or Fisher's exact test (2 × 2 comparisons). Numerical data were assessed for outliers and normality using the Shapiro–Wilk test, Q–Q plots and histograms. Next, depending on variance homogeneity (Levene's test), group comparisons were conducted using Student's independent *t*‐test, Welch's test (normally distributed data) or the Mann–Whitney *U*‐test (non‐normally distributed data). Quade's test, a non‐parametric alternative for analysis of covariance, was performed to adjust for daylight time as a proxy for season, for the physical activity analysis (Demeyer et al., 2014). Comparison of numerical data within groups was performed after outlier assessment and normality testing of the calculated difference (Δ_mBCST‐CPET_), inspection of Q–Q plots and histograms. Next, either Student's paired *t*‐test (normally distributed data) or a Wilcoxon rank test (non‐normally distributed dated) was used. For numerical data, mean or median differences with 95% confidence interval (CI) are reported.

An exploratory analysis examined the greater heterogeneity in relative mBCST workload in people with COPD using Spearman rank correlation analysis and a subgroup analysis based on limitation to exercise (abnormal exercise response with ventilatory limitation vs. normal exercise response). Correlation strength was interpreted following guidelines of Hopkins et al. (2009). A two‐sided α‐level of 0.05 was used for statistical significance.

A sample size was not calculated, because the present study is part of a cross‐over trial (ClinicalTrials.Gov #NCT05874999) (Jakobsson et al., 2025). Within this cross‐over trial, in which exercise intensity and plasma brain‐derived neurotrophic factor are the primary outcomes, a sample size calculation (*n* = 16 per group) was performed, which we deem sufficient for the present study (Julious, 2005).

## 3 RESULTS

### 3.1 Participants

**FIGURE 2:**
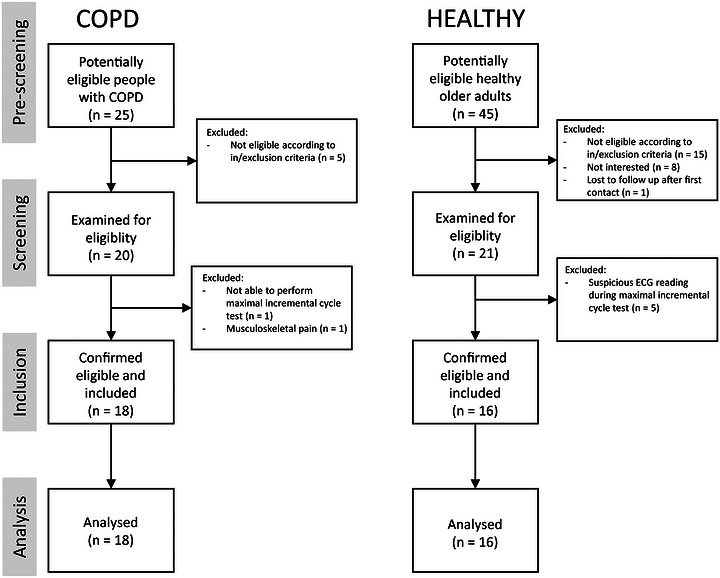
Flowchart of participants. Abbreviation: COPD, chronic obstructive pulmonary disease; ECG, electrocardiography.

Figure 2 depicts the flow of participants from screening to analysis.

#### 3.1.1 Participant characteristics

People with COPD were 75 ± 6 years old, and 56% were female, which was similar for age‐, sex‐ and physical activity‐matched healthy older adults. Most people with COPD were ex‐smokers (88.9%) and classified as GOLD A (22.2%) or B (66.7%) (for more details, see Table 2).

**TABLE 2:** Participant characteristics.

Characteristics	COPD (*n* = 18)	Healthy older adults (*n* = 16)	*p*‐value
**General characteristics**			
Age (years)	75 (6)	74 (5)	0.748
Sex (female; *n* [%])	10 [55.6]	8 [50.0]	1.000^f^
BMI (kg/m^2^)	24.8 (3.6)	25.2 (2.1)	0.659
Fat mass (%)	30.2 (8.5)	28.7 (7.8)	0.586
FFMI (kg/m^2^)	17.2 (2.5)	17.9 (1.6)	0.343
Smoking status (non‐smoker, ex‐smoker, smoker (*n* [%])	1 [5.6], 16 [88.9], 1 [5.6]	9 [56.3], 7 [43.8], 0 [0.0]	**0.004^g^**
Pack‐years (ex‐smoker, smoker)^a^	26 (14), 56 [Correction added on 17th October 2025 after first online publication: This value has been updated]	12 (12), 0 (0) [Correction added on 17th October 2025 after first online publication: This value has been updated]	**0.049** [Correction added on 17th October 2025 after first online publication: This value has been updated]
Hospitalization < 12 months (0, 1, ≥ 2; *n* [%])	14 [77.8], 2 [11.1], 2 [11.1]	16 [100.0], 0 [0.0], 0 [0.0]	0.133^g^
Non‐respiratory hospitalizations (0, 1, ≥ 2; *n* [%])	15 [83.3], 2 [11.1], 1 [5.6]	16 [100.0], 0 [0.0], 0 [0.0]	0.232^g^
Respiratory hospitalizations (0, 1, ≥ 2; *n* [%])	16 [88.9], 2 [11.1], 0 [0.0]	16 [100.0], 0 [0.0], 0 [0.0]	0.487^f^ [Correction added on 17th October 2025 after first online publication: “f” has been added as superscript to the value “0.487.”]
AECOPD hospitalizations (0, 1, ≥ 2; *n* [%])	17 [94.4], 1 [5.6], 0 [0.0]	–	–
AECOPD (0, 1, ≥ 2; *n* [%])	15 [83.3], 2 [11.1], 1 [5.6]	–	–
CAT score (points)	15 (6)	–	–
mMRC dyspnoea (points)	2 (1–2)	1 (0–1)	**0.003**
GOLD (ABE; *n* [%])^b^	4 [22.2], 12 [66.7], 2 [11.1]	–	–
**Lung function**			
FEV_1_ (L)	1.75 (0.40)	2.77 (0.51)	**<0.001**
FEV_1_ (% predicted)	71.5 (15.7)	106.5 (13.4)	**<0.001**
GOLD (1–4; *n* [%])	6 [33.3], 10 [55.6], 2 [11.1], 0 [0.0]	–	–
FVC (L)	3.23 (0.74)	3.80 (0.73)	**0.030**
FVC (% predicted)	99.5 (14.8)	110.6 (11.1)	**0.020**
FEV_1_/FVC	0.54 (0.09)	0.73 (0.04)	**<0.001**
IC (L)	2.39 (0.56)	2.78 (0.48)	**0.039**
TLC (L)	6.01 (1.02)	6.27 (1.25)	0.515
TLC (% predicted)^c^	104.6 (14.3)	101.6 (7.0)	0.478
FRC (L)	3.75 (3.41–4.46)	3.43 (2.85–4.59)	0.237
FRC (% predicted)^c^	126.0 (28.9)	104.5 (16.8)	**0.019**
FRC/TLC	66.0 (6.7)	58.2 (6.5)	**0.002**
RV (L)	2.64 (0.57)	2.34 (0.61)	0.145
RV (% predicted)^c^	120.7 (94.0–129.1)	96.4 (81.5–110.1)	**0.015**
RV/TLC	43.8 (40.2–46.0)	37.7 (32.9–41.1)	**<0.001**
RV/TLC (% predicted)^c^	114.6 (19.4)	94.2 (10.9)	**0.001**
DLCO SB (mmol/min/kPa)	5.04 (1.52)	6.77 (1.32)	**0.001**
DLCO SB (% predicted)^d^	70.0 (16.9)	92.1 (16.1)	**<0.001**
**Medication use**			
Number of inhalers/lung medication (*n*)	2 (1–3)	–	–
Short‐acting only (*n* [%])	1 [5.6]	–	–
Short‐ + long‐acting (*n* [%])	4 [22.2]	–	–
Long‐acting only (*n* [%])	5 [27.8]	–	–
Short‐ + long‐acting + ICS (*n* [%])	5 [27.8]	–	–
Long‐acting + ICS (*n* [%])	3 [16.7]	–	–
Phosphodiestarese‐4 inhibitors (*n* [%])	2 [11.1]	–	–
β‐Blockers (*n* [%])	7 [38.9]	2 [12.5]	0.125^f^
Other heart medication (*n* [%])	14 [77.8]	8 [50.0]	0.151^f^
Cholesterol medication (*n* [%])	6 [33.3]	5 [31.3]	1.000^f^
Anti‐coagulation or anti‐aggregation medication (*n* [%])	7 [38.9]	2 [12.5]	0.125^f^
Total number of medications (*n*)	6.4 (2.9)	1.8 (1.5)	**<0.001**
**Physical activity**			
Step count (steps/day)^e^	5458 (4040–9726)	8266 (6145–11852)	0.055^h^
MVPA (min/day)^e^	5 (0–34)	23 (20–53)	0.075^h^

*Note*: Numerical data are expressed as the mean (SD) or median (quartile 1–quartile 3) depending on normality of data distribution, and categorical data are expressed as number [percentage]. Comparison of numerical data between groups was performed via Student's independent *t*‐test or Welch's test (normal data distribution) or Mann–Whitney *U*‐test (non‐normal data distribution). Bold *p*‐value indicates significant difference between people with COPD and healthy older adults using a two‐sided α‐level of 0.05 for statistical significance.

Abbreviations: AECOPD, acute exacerbation of COPD; BMI, body mass index; CAT, COPD assessment test; COPD, chronic obstructive pulmonary disease; DLCO SB, diffusion capacity of the lung for carbon monoxide‐single breath; FEV_1_, forced expiratory volume in 1 s; FFMI, fat‐free mass index; FRC, functional residual capacity; FVC, forced vital capacity; GOLD, global initiative for chronic obstructive lung disease; IC, inspiratory capacity; mMRC, modified medical research council; MVPA, moderate to vigorous physical activity; RV, residual volume; TLC, total lung capacity.

^a^No SD reported due to sample size: *n* = 1. Statistical comparison between groups only performed for ex‐smokers.

^b^CAT score is used as symptom‐scoring instrument to determine GOLD ABE category.

^c^Altered sample size (COPD, *n* = 15; healthy, *n* = 15) because there were no Global Lung Initiative reference values for persons >80 years old.

^d^Altered sample size (COPD, *n* = 17) because there were no Global Lung Initiative reference values for persons >85 years old.

^e^Altered sample size (COPD, *n* = 16; healthy, *n* = 15) due to missing data because of technical error or not wearing an accelerometer.

^f^Fisher's exact test.

^g^χ^2^ test for homogeneity.

^h^Quade's test, a non‐parametric alternative for analysis of covariance, was performed to adjust for daylight time, which is a proxy for season, for the physical activity analysis.

#### 3.1.2 Maximal exercise capacity

All participants performed a maximal‐effort CPET, with an average duration of 523 ± 162 s (∼9 min) for people with COPD and 642 ± 73 s (∼11 min) for healthy older adults (Tables S2 and S3). [Correction added on 17th October 2025 after first online publication: The duration has been updated to 11 min from 10 min.] People with COPD mainly reported breathlessness (39%) or leg fatigue (44%) as the reason for stopping, whereas leg fatigue was the most common reason in healthy older adults (60%) (Table S2). Maximal exercise capacity was significantly reduced in people with COPD in comparison to healthy older adults (Table S3). Detailed data on ventilatory, gas exchange, cardiovascular, metabolic response and symptom ratings are presented in Table S3.

### 3.2 mBCST

#### 3.2.1 Feasibility

**FIGURE 3:**
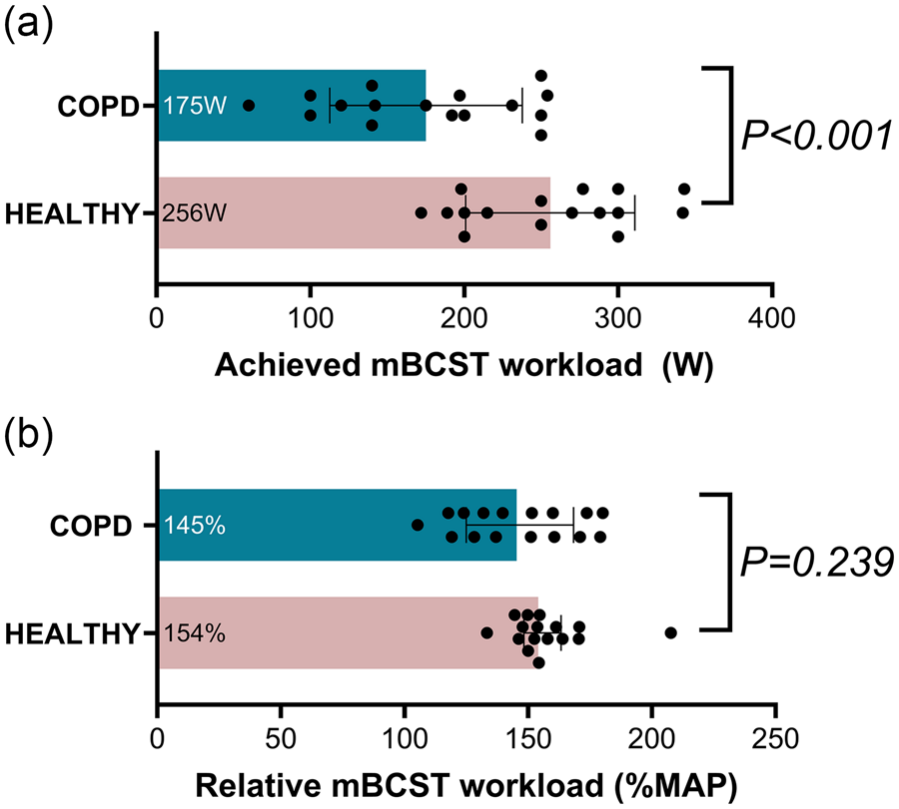
Achieved mBCST workload (a) and relative mBCST workload (b) of people with COPD (*n* = 16; blue bar) and healthy older adults (*n* = 16; pink bar). (a) A Student's independent *t*‐test was performed; the mean ± SD are indicated by bar height and error bars, and the mean is provided on the left side of the bar. (b) A Mann–Whitney *U*‐test was performed; the median and interquartile range are indicated by bar height and error bars, and the median is provided on the left side of the bar. Abbreviations: COPD, chronic obstructive pulmonary disease; mBCST, modified Borg cycle strength test; MAP, maximal aerobic power.

All participants that completed the mBCST [Correction added on 17th October 2025 after first online publication: The word “participants” has been replaced with “participants that completed the mBCST.”] reached supramaximal intensities during the mBCST [COPD, 145 (125–168) %MAP; and older adults healthy, 154 (148–163) %MAP; Table 1; Figure 3b]. Feasibility was assessed according to the test feasibility framework (Table 1). Concerning familiarity required, all participants completed the single familiarization bout except for one person with COPD who could not reach the target RPM. Regarding completion complexity, the mBCST was completed by all healthy older adults and 16 of 17 persons with COPD. The total duration, including warm‐up and cool‐down, was 659 ± 45 s (∼11 min) for completed tests.

Test administrators experienced two conduction problems (6%), and the scoring complexity was low in 56% of tests, because RPE was used as the stop‐criterion, inferring that the workload of the last step of the mBCST is the achieved mBCST workload. When RPM was the stop‐criterion, an additional calculation step was required to determine the achieved mBCST workload (see mBCST template in Supporting Information; Table 1). Interpretability, cost and safety aspects of the mBCST are described in a narrative form in Table 1.

#### 3.2.2 mBCST external exercise intensity

**FIGURE 4:**
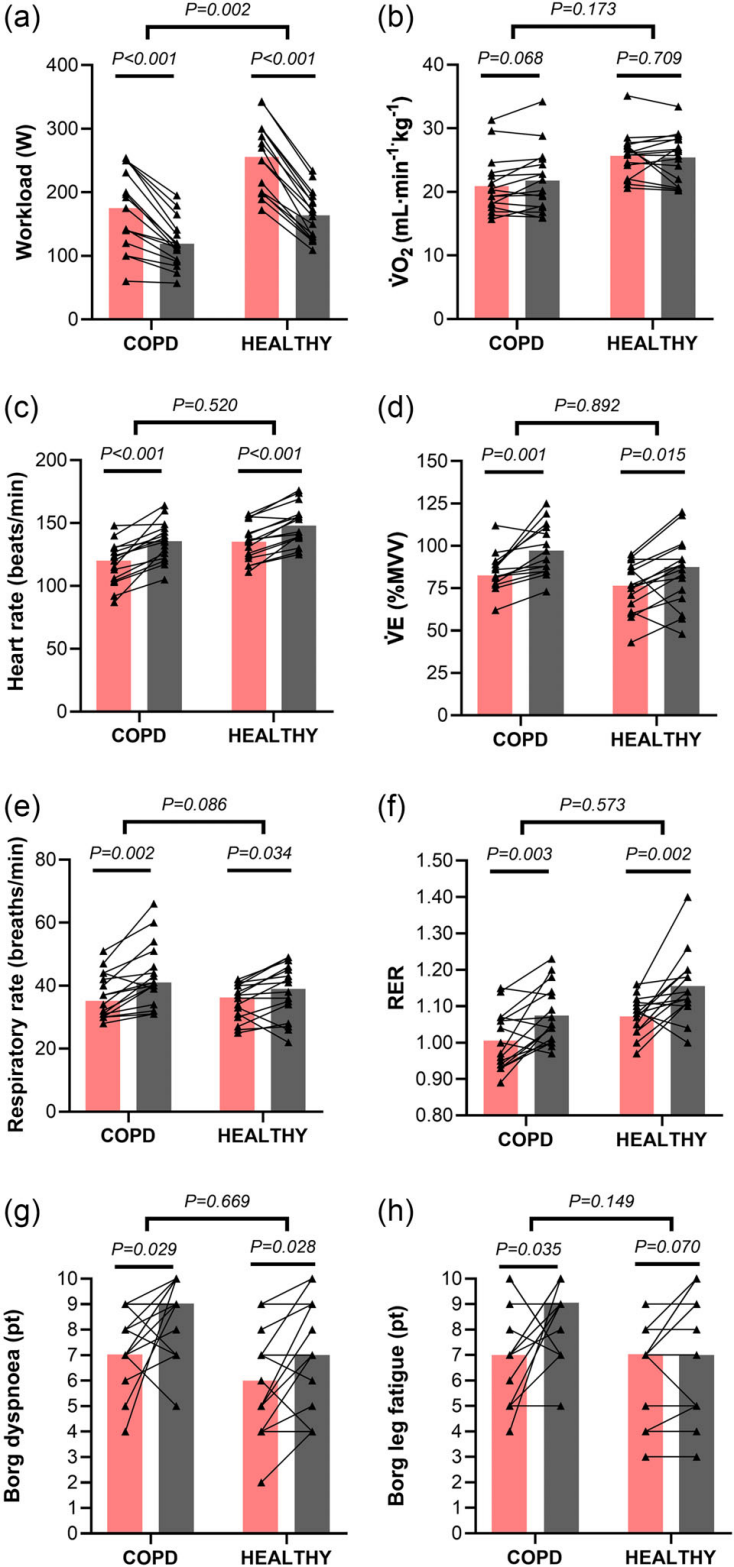
Physiological response and symptoms at end‐exercise of mBCST (pink bar) and CPET (grey bar) performed in people with COPD (*n* = 16) and healthy older adults (*n* = 16). Individual data and measures of central tendency are shown. The use of parametric or non‐parametric statistical analysis was based on the normal distribution of the data; therefore, bar charts in (a, b, f) depict the mean, whereas bar charts in (c–e, g, h) depict median. Lines between mBCST and CPET show the exact *p*‐value of the within‐group analysis (Student's paired *t*‐test or Wilcoxon rank test), while the ticked down lines show the exact *p*‐value of the between‐group analysis on the ΔmBCST–CPET value (Student's independent *t*‐test or Welch's test, or Mann–Whitney *U*‐test). (c, d) There is a altered sample size (COPD, *n* = 15). Abbreviations: COPD, chronic obstructive pulmonary disease; CPET, cardiopulmonary exercise test; mBCST, modified Borg cycle strength test; MVV, maximal voluntary ventilation obtained from MVV manoeuvre during lung function testing; RER, respiratory exchange ratio; ${\dot{V}}_{E}$, ventilation; ${\dot{V}}_{O_{2}}$, rate of oxygen consumption.

The achieved mBCST workload was significantly higher in comparison to the MAP in both groups, with people with COPD having a significantly lower increase in absolute workload (Δ*W*_mBCST‐CPET_) in comparison to healthy older adults (Figure 4a; Table S4). However, expressed relative to MAP, the mBCST workload was not different between both groups (Figure 3b).

#### 3.2.3 mBCST physiological response

At isoMAP, all parameters and symptom ratings were significantly lower compared with end‐CPET in both groups, except for V˙E/V˙EV˙CO2V˙CO2 in people with COPD and oxygen pulse in healthy older adults (Table S5).

The ${\dot{V}}_{O_{2}}$ at end‐mBCST did not differ from end‐CPET in either group (Figure 4b; Table S4). In contrast, cardiovascular (HR and systolic blood pressure) and ventilatory responses were significantly lower at end‐mBCST compared with end‐CPET [Correction added on 17th October 2025 after first online publication: The term “CPET” has been updated to “end‐CPET.”], whereas the oxygen pulse was significantly higher (Figure 4c–e; Table S4). Other gas exchange and metabolic parameters are reported in Figure 4f and Table S4. Notably, ratings of perceived exertion, breathlessness and leg fatigue at end‐mBCST were significantly reduced compared with end‐CPET in both groups, except for the end‐mBCST rating of leg fatigue in healthy older adults, which showed no difference (Figure 4g‐h [Correction added on 17th October 2025 after first online publication: The figure citation has been updated to “Figure 4g‐h” from “Figure 1,2,4.”]; Table S4).

No difference between people with COPD and healthy older adults was observed in the mean or median difference (Δ_mBCST‐CPET_ at isoMAP or end‐exercise) of ventilatory, gas exchange, cardiovascular and metabolic parameters or symptom ratings, except for gas exchange at isoMAP (Figure 4b–f; Table S4 and S5). Likewise, when expressing end‐mBCST parameters relative to CPET, no differences were observed between groups (Table S6).

#### 3.2.4 Exploration of the heterogeneous results in relative mBCST workload in people with COPD

**FIGURE 5:**
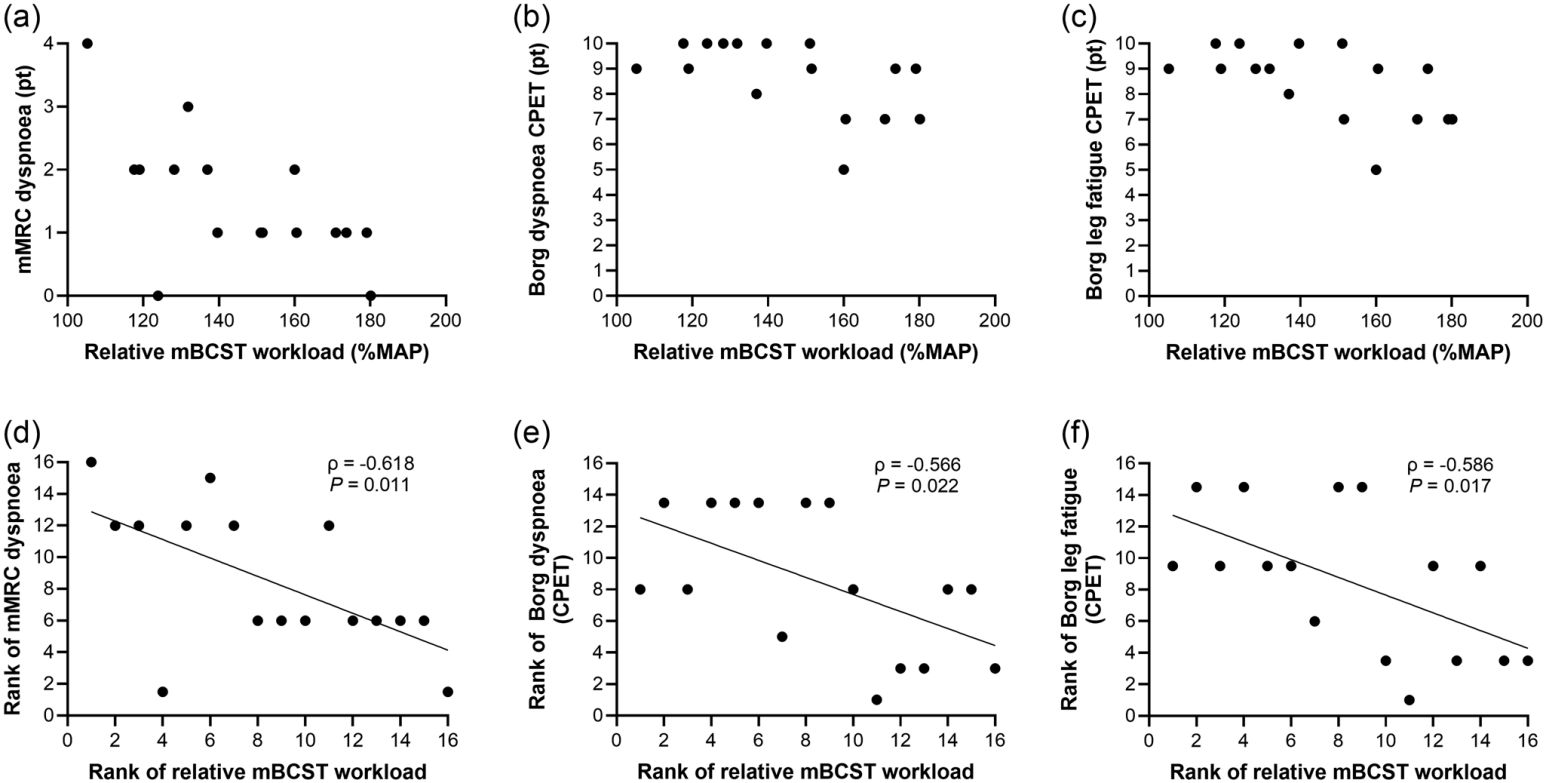
Associations between relative mBCST workload and mMRC dyspnoea, Borg dyspnoea CPET and Borg leg fatigue CPET in people with COPD (*n* = 16) depicted via scatter plots. Spearman rank correlation was performed. For correct visualization and interpretation of the Spearman rank correlation, (a–c) shows the scatter plots of the original data, whereas (d–f) shows the scatter plots of the original data transformed into ranks. Abbreviations: COPD, chronic obstructive pulmonary disease; CPET, cardiopulmonary exercise test; MAP, maximal aerobic power; mBCST, modified Borg cycle strength test; mMRC, modified Medical Research Council.

Although relative mBCST workload did not differ between people with COPD and healthy older adults, Figure 3 shows a greater heterogeneity in people with COPD. Association analyses showed that achieving a higher relative mBCST workload was moderately to strongly associated with a lower modified Medical Research Council (mMRC) dyspnoea score (ρ = −0.618; *p* = 0.011; Figure 5a, d), higher diffusion capacity (ρ = 0.575; *p* = 0.025), and more minutes per day performing moderate to vigorous physical activity (ρ = 0.513; *p* = 0.042). Non‐significant moderate associations were observed with total number of medications, daily step count and hyperinflation (functional residual capacity/total lung capacity) (Table S7). Likewise, association analyses of CPET parameters showed that a higher relative mBCST workload was moderately to strongly associated with a lower V˙E/V˙EV˙CO2V˙CO2 nadir (ρ = −0.588; *p* = 0.017) and a a lower score on the Borg dyspnoea scale (ρ = −0.566; *p* = 0.022; Figure 5b, e) and Borg leg fatigue scale (ρ = −0.586; *p* = 0.017; Figure 5c, f). Non‐significant moderate associations were observed with ${\dot{V}}_{O_{2}}$ peak and time to exhaustion (Table S8).

**FIGURE 6:**
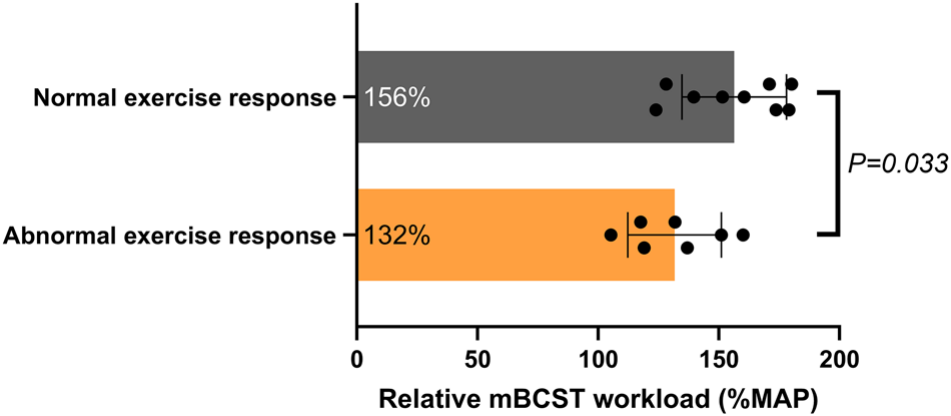
Subgroup analysis based on limitation to exercise in people with COPD (*n* = 16) during CPET. Relative mBCST workload is compared between people with COPD who have a normal exercise response (grey bar; *n* = 9) and people with COPD who have an abnormal exercise response with ventilatory limitation (orange bar; *n* = 7). A Student's independent *t*‐test was performed; the mean ± SD are depicted by bar height and error bars, and the mean is provided on the left side of the bar. Abbreviations: COPD, chronic obstructive pulmonary disease; CPET, cardiopulmonary exercise test; MAP, maximal aerobic power; mBCST, modified Borg cycle strength test.

Subgroup analysis based on limitation to exercise indicated that people with COPD who had an abnormal exercise response (i.e., peak ${\dot{V}}_{O_{2}}$ or MAP of <85% of predicted) with ventilatory limitation had a significantly lower relative mBCST workload than those with a normal exercise response [mean difference (95% CI) = 25 (2–47) %MAP; Figure 6].

## 4 DISCUSSION

The mBCST proved to be feasible, achieving median supramaximal exercise intensities close to 150% of MAP (range 105%–208% of MAP). Notably, this was accomplished with a similar physiological demand in both people with COPD and age‐, sex‐ and physical activity‐matched healthy older adults. Importantly, the mBCST enabled supramaximal workloads while remaining a submaximal test, as demonstrated by its lower physiological response in comparison to CPET, eliminating the need for maximal effort.

### 4.1 Feasibility

Most importantly, the objective and practical meaning (interpretability) of using the mBCST in our study was to use the achieved workload to prescribe SupraHIIT (Frykholm et al., 2024; Hedlund et al., 2019; Simonsson et al., 2023). Achieved mBCST workloads were indeed supramaximal (±150% of MAP), enabling the successful prescription of a feasible SupraHIIT session for both healthy older adults and individuals with COPD (Jakobsson et al., 2025). Following the exercise training principle of specificity, exercise prescription is most accurate when based on physiological anchors closely aligned with the targeted exercise domain. Therefore, we consider mBCST‐derived intensities to be better suited than MAP‐derived intensities when prescribing SupraHIIT. Further research comparing the effectiveness of SupraHIIT prescription based on mBCST‐ or MAP‐derived intensities is, however, warranted to confirm this claim.

The mBCST has previously been used to prescribe SupraHIIT in healthy older adults (Frykholm et al., 2024; Hedlund et al., 2019; Simonsson et al., 2023), suggesting its feasibility in this population. Our detailed feasibility analysis, based on a published framework (Robertson et al., 2017), confirms this assumption, because every healthy older adult completed the mBCST within 10–12 min (including rest and recovery), with low assessor scoring complexity. In people with COPD, feasibility was almost identical compared with healthy older adults, with 94% completing the mBCST. However, we need to acknowledge that the high target cadence (80–90 RPM) might be challenging to reach for some people with COPD, because one study participant failed the familiarization bout. Whether this is attributable to being cycling ‘naive’ or reduced lower‐limb muscle power, common in older adults and people with COPD (Reid & Fielding, 2012; Roig et al., 2010; Tanguay et al., 2023), remains to be determined. More importantly, acknowledging the ability of a person to reach a sufficiently high cadence is a prerequisite for performing the mBCST when the aim is to use the results for the prescription of SupraHIIT, where higher cadences facilitate short‐duration intervals against a very high external resistance (Hedlund et al., 2019). Hence, the performance of a familiarization bout is recommended when performing the mBCST.

Overall, the mBCST scores well on the feasibility components of scoring complexity, completion complexity, duration and safety. Another important component of feasibility is costs. Specifically, equipment cost remains a limitation, because the mBCST per our methodology requires a cycle ergometer capable of rapidly reaching high loads, which is often available in research or clinical centres but less so in rehabilitation centres or public gyms, because these ergometers tend to be expensive. An alternative approach, demonstrated to be feasible in previous research where the individuals performed the mBCST on a spinning bike where external resistance can rapidly be adjusted manually by turning a knob (Frykholm et al., 2024; Hedlund et al., 2019; Simonsson et al., 2023), could be used. Importantly, this approach assumes knowledge of the relationship between power output and braking force at the given cadence (80–90 RPM), which must be accounted for to ensure accurate test performance.

### 4.2 Physiological response to mBCST

To our knowledge, this is the first study to determine the physiological response to the mBCST in healthy older adults and people with COPD. In line with the purpose of Borg (Borg, 1982), a submaximal physiological response was observed when performing the mBCST. Values of all physiological parameters, except workload, ${\dot{V}}_{O_{2}}$ and thus oxygen pulse (${\dot{V}}_{O_{2}}$/HR), and V˙E/V˙EV˙CO2V˙CO2 were significantly lower at isoMAP and end‐mBCST compared with CPET. The physiological demand of the mBCST ranged from 75% to 105% (people with COPD) and from 79% to 110% (healthy older adults) of CPET, dependent on the physiological parameter, which is in line with the mBCST termination criteria of RPE ≥ 17, corresponding to vigorous intense exercise (Garber et al., 2011).

Achieved absolute mBCST workloads were lower in people with COPD compared with age‐, sex‐ and physical activity‐matched healthy older adults, but similar when expressed relative to MAP (∼150% of MAP) achieved during CPET. Additionally, the physiological response to the mBCST, relative to CPET, did not differ between groups, suggesting that anaerobic cycling capacity might be preserved in people with COPD. Healthy older adults demonstrated a consistent mBCST workload/MAP ratio [median (quartile 1–quartile 3) = 154 (148–163) %MAP], whereas greater heterogeneity was observed in people with COPD [median (quartile 1–quartile 3) = 145 (125–168) %MAP]. Further analysis showed that a lower mBCST workload relative to MAP was associated with more breathlessness in daily life, lower diffusion capacity, lower physical activity and having an abnormal exercise response with ventilatory limitation in people with COPD. This finding indicates that the mBCST workload/MAP ratio seems distorted in a subgroup of highly symptomatic and inactive people with COPD, suggesting that their anaerobic power reserve might be reduced.

This should be investigated further, because prescription of SupraHIIT based on these achieved mBCST workloads might lead to suboptimal training prescription and results in those individuals. Alternative tests that circumvent the disabling feature of breathlessness might deliver better results for these individuals. For example, the steep ramp anaerobic test (SRAT) has been used in various clinical populations to personalize HIIT, for example, in chronic heart failure and COPD (Meyer et al., 1997; Puhan et al., 2006; Trul‐Kreuze et al., 2024). During the SRAT, people with COPD reached >2‐fold higher workloads compared with CPET (Chura et al., 2012). Additionally, the SRAT is extremely short (∼1 min) (Chura et al., 2012), whereas mBCST and CPET duration in our study were ∼5 and ∼9 min, respectively. Intuitively, it would be hypothesized that the very short duration of the SRAT would lead to lower ventilatory effort (Trul‐Kreuze et al., 2024). This, however, was not observed in people with COPD, because the ventilatory effort at the end of SRAT was similar in comparison to the end of CPET (Chura et al., 2012). A study comparing achieved workloads and physiological response during CPET, mBCST and SRAT is needed to provide a better understanding of where the achieved mBCST (∼MPO30) and SRAT workloads are positioned on the power–duration curve. Eventually, these findings could also guide investigation into which exercise test is most suited for which individual and for the most optimal prescription of SupraHIIT in people with COPD, particularly in those with a distorted mBCST workload/MAP ratio.

Within this context, we should also not overlook the impact of lower‐limb muscle dysfunction (Maltais et al., 2014) and peripheral muscular limitation to exercise often experienced by people with COPD (Hamilton et al., 1995; Killian et al., 1992), because cycling peak power output is known to be reduced (Yquel et al., 2006), and supramaximal cycling efforts are strongly correlated with quadriceps muscle function in people with COPD (Butcher et al., 2012). In addition, other contributory factors, such as fear of exercise or psychological barriers, which are common in people with COPD, potentially impact their ability or willingness to engage in high‐intensity efforts (Vardar‐Yagli et al., 2019; Wang et al., 2023).

### 4.3 Limitations and implications for future research

Although the mBCST seems to be a feasible exercise test that enables supramaximal external exercise intensity in healthy older adults and people with COPD, some limitations regarding our study methodology should be acknowledged and addressed in future research.

Within our study, mBCST workload step increments were based on the achieved MAP during CPET. This implies that a CPET is necessary before performing a mBCST. In theory, for healthy individuals, step increments could also be based on estimated MAP values via normative values or reference equations. For people with COPD or other chronic respiratory diseases, who experience a negative impact on maximal exercise capacity, workload increments for a CPET of ∼10 min could be estimated using equations found in the ERS CPET guidelines to provide a rough estimation of MAP (Radtke et al., 2019). The mBCST has mostly been used as an exercise test that enables supramaximal intensities, and therefore can be used to prescribe SupraHIIT (Frykholm et al., 2024; Hedlund et al., 2019; Simonsson et al., 2023). However, conducting a CPET before an mBCST is still recommended for ensuring that individuals, especially those with chronic conditions, can safely perform vigorous exercise training and to prescribe SupraHIIT better, because the lower limit of the anaerobic power reserve, MAP, will be known.

It should be noted that data, expressed in absolute values and relative to CPET, for some physiological variables measured via indirect calorimetry, a sphygmomanometer and pulse oximetry, needs to be interpreted with caution, because different equipment was used during the mBCST and CPET. However, this was the same for every study participant, and as such, it does not impact findings related to comparisons between people with COPD and age‐, sex‐ and physical activity‐matched healthy adults. For follow‐up studies, we recommend using the same equipment for both mBCST and CPET to eliminate this source of bias.

We also performed IC manoeuvres during the CPET and mBCST, but we were not able to use the CPET IC data due to a large amount of invalid data. Therefore, it was not possible to identify people with COPD who suffer from dynamic hyperinflation during CPET and whether those people would suffer from equal or less dynamic hyperinflation during the mBCST. Indeed, given that inspiratory constraint is an important contributor to ventilatory limitation to exercise in people with COPD (Neder et al., 2019), a larger‐scale investigation incorporating inspiratory constraint assessment is warranted to gain a better understanding of the physiological response of people with COPD during mBCST and clarify its relationship with mBCST performance.

Another limitation of our study is the relatively small sample size of individuals with COPD, most of whom had only mild to moderate airflow obstruction and relatively preserved exercise capacity. This is reflected in their mean peak ${\dot{V}}_{O_{2}}$ of 93% ± 18% of predicted, V˙E/V˙EV˙CO2V˙CO2 nadir of 32.4 (quartile 1–quartile 3: 30.5–34.7), and peripheral oxygen saturation of 97% (quartile 1–quartile 3: 95%–98%). However, considering individual variability, half of our COPD sample (9 of 18) exhibited abnormal exercise performance. Among these individuals, the mBCST was still feasible in 78% of people with COPD, supporting its applicability even in those with greater impairment. Nonetheless, as discussed earlier, the potential for a distorted mBCST workload/MAP ratio in a subset of highly symptomatic, inactive people with COPD still suggests reduced applicability among those with more severe impairments. This highlights the need for further research including a wider spectrum of people with COPD to gain a better understanding of its applicability across the entire COPD spectrum.

Lastly, next to feasibility, other measurement properties of the mBCST, such as reliability, validity and responsiveness, still need to be determined before the mBCST could be implemented in practice (Robertson et al., 2017).

## 5 CONCLUSION

The mBCST is feasible in people with COPD and in age‐, sex‐ and physical activity‐matched healthy older adults. It enables supramaximal external exercise intensities of >150% of MAP obtained during CPET and might, therefore, be considered to be better suited in comparison to MAP‐derived intensities when selecting an exercise test for prescribing supramaximal exercise. Additionally, the physiological response to the mBCST in people with COPD is similar to that of healthy older adults, and the mBCST is accomplished with a lower physiological response compared with CPET, eliminating the need for maximal effort. Future research should refine the application of the mBCST and validate its measurement properties to improve exercise‐based interventions for people with COPD.

## AUTHOR CONTRIBUTIONS

Study conception and design: Jana De Brandt, Johan Jakobsson, Mattias Hedlund and André Nyberg. Data acquisition: Jana De Brandt, Johan Jakobsson, Mattias Hedlund, Thomas Sandström and André Nyberg. Analysis and interpretation: Jana De Brandt, Johan Jakobsson and André Nyberg. Drafting of manuscript: Jana De Brandt. Critical revision of manuscript: Jana De Brandt, Johan Jakobsson, Mattias Hedlund, Thomas Sandström and André Nyberg. All authors approved the final version of the manuscript and agree to be accountable for all aspects of the work in ensuring that questions related to the accuracy or integrity of any part of the work are appropriately investigated and resolved. All persons designated as authors qualify for authorship, and all those who qualify for authorship are listed.

## CONFLICT OF INTEREST

J.D.B., J.J., M.H., T.S., and A.N. have no conflict of interest to declare.

## Supporting information



Supporting Information: more details on method section and additional results (Table S1‐S8)

## Data Availability

The data that support the findings of this study are available from the corresponding author upon reasonable request.
