# Supplementary material for: The modified Borg cycle strength test (mBCST): Feasibility and physiological response in people with COPD and healthy older adults
Source: Exp Physiol. 2025 Jul 10;111(6):3010–25. doi: 10.1113/EP092151 (PMC13238777; doi:10.1113/EP092151)
Supplement: Supplementary file 1 — Supporting Information: more details on method section and additional results (Table S1‐S8) [file EPH-111-3010-s001.docx]

# **Supporting information**

**The modified Borg cycle strength test (mBCST): feasibility and physiological response in people with COPD and healthy older adults**

De Brandt J*, Jakobsson J*^1^, Hedlund M^1^, Sandström T^2^, Nyberg A^1^.

^1^Faculty of Medicine, Department of Community Medicine and Rehabilitation, Section of Physiotherapy, Umeå University, Umeå, Sweden

^2^Faculty of Medicine, Department of Public Health and Clinical Medicine, Umeå University, Umeå, Sweden

*shared first authorship

**Methods**

### Participants instructions and recruitment pathways

**Instructions:** Participants were requested to adhere to the following guidelines: maintain usual medication use and abstain from i) strenuous physical activity within 48 hrs; ii) alcohol consumption within 24 hrs; iii) caffeine intake within 6 hrs; and iv) smoking within 8 hrs of the study visit.

**Recruitment pathways:** People with COPD were recruited via two pathways: i) referrals by a COPD nurse at the University Hospital of Umeå; and ii) previous study participants who had consented to future contact were contacted by the research team. Healthy older adults were recruited via advertisement.

### Participant characteristics

Age, sex, hospitalization history, exacerbation history (COPD-specific), medication intake, smoking status and packyears, COPD assessment test (CAT) (Jones et al., 2009), and modified Medical Research Council (mMRC) scale for dyspnoea (Stenton, 2008) were obtained. Body height was assessed by a calibrated stadiometer. Body composition assessment was performed via bio-electrical impedance analysis (BC-418MA, Tanita, Tokyo, Japan) by placing the feet on the dedicated area on the weight scale, standing upright and holding a handle in each hand with straight arms during the measurement. Body weight, body mass index, fat-free mass, fat-free mass index (fat-free mass/height^2^) and fat-mass were obtained. Lung function testing consisted of forced spirometry (Miller et al., 2005), slow spirometry (Miller et al., 2005), static and dynamic lung volumes (Wanger et al., 2005) and diffusion capacity according (Graham et al., 2017) to European Respiratory Society (ERS) and American Thoracic Society (ATS) guidelines. Absolute and percent predicted values are reported. The Global Lung Function Initiative calculator (<https://gli-calculator.ersnet.org/>) was used to calculate percent predicted values.

### Physical activity assessment

Steps per day and moderate to vigorous physical activity (MVPA) were objectively measured via a portable accelerometer (Movemonitor, McRoberts, The Hague, The Netherlands). The accelerometer was worn for seven consecutive days, except for water-based activities, on the lower back via a stretchable belt with Velcro strap. Accelerometer data consisting of ≥8h/day wear time with ≥4 days of measurements, excluding test days, were considered valid, and the median number of steps on valid days was used as matching parameter (Demeyer et al., 2021).

### Cardiopulmonary exercise test

The CPET was performed on a stationary electronically-braked ergometer (Rodby RE990, Rodby Innovation AB, Vänge, Sweden) according to ERS guidelines (Radtke et al., 2019). Participants were equipped with a facemask (Vyaire, Mettawa, IL, USA), to perform gas sampling and volume transduction for the measurement of inspired and expired gas volumes and airflow variables using indirect calorimetry via a metabolic cart with breath-by-breath technology (Vyntus CPX, Vyaire, Mettawa, IL, USA), a 12-lead electrocardiography (ECG; Cardiolex, Solna, Sweden) and a forehead sensor for peripheral oxygen saturation (8000R, Vyaire, Mettawa, IL, USA). Gas calibration and automatic volume calibration of the metabolic cart was performed before every test. The maximal flow-volume loop, which is needed to perform exercise flow-volume loops to establish operational lung volumes (Guenette et al., 2013), was obtained from the aforementioned performed forced spirometry. Participants rested for three minutes on the bike, followed by three minutes of warm-up at the lowest watt (20 W) at a pedalling cadence of 50 to 70 revolutions per minute (RPM), a test phase with an incremental ramp protocol at a pedalling cadence of 50 to 70 RPM, and three minutes of cool-down at the lowest watt at a slower pedalling cadence of 30 to 50 RPM. The choice of starting workload (20W) and ramp protocol (7 to 20W/min) was guided by normative values (based on age, sex, height, disease severity (only for COPD), physical activity and estimated exercise capacity based on consultation with the participant) for expected MAP. Manual blood pressure, rate of perceived exertion (RPE) and rating of dyspnoea and leg fatigue (Borg CR10) were obtained in the last minute of rest, warm-up, every other minute during the test-phase, at the end of the test-phase, and in the last minute of cool-down. Inspiratory capacity manoeuvres were performed every minute during rest, in the last minute of warm-up, every other minute during the test-phase, just before the end of the test-phase, and in the last minute of cool-down (Guenette et al., 2013). Reasons for test termination were recorded immediately after stop. As a safety measure, the Borg CR10 scale to assess chest pain was used when indicated (this measure is not an outcome variable). Maximal encouragement was given during the test.

### modified Borg cycle strength test: history, participant instructions and monitoring

**History of mBCST:** Originally, the BCST was described by Gunnar Borg and tested in healthy, young adults using a starting load and incremental step sizes of 50W (Borg, 1982). Hedlund et al. later adapted this protocol by adding two protocols (36W and 45W) with lower starting load and incremental steps to suit healthy older adults as at least three steps are preferred to perform the data-extrapolation (Hedlund et al., 2019). In this study, we have further modified the protocol for people with COPD, using starting loads and incremental steps (maximum 10 steps) ranging from 15 to 50W. For SupraHIIT prescription, initial methods relying on extrapolation of heart rate and rating of perceived exertion (RPE) encountered issues with overestimation of maximum mean power output over 30 seconds (MPO30) (Hedlund et al., 2019). Hence, an alternative methodology was proposed, using the highest workload achieved during the BCST directly to estimate maximum MPO30. This approach reduced the risk of overestimation, and was later applied and better suited to prescribe SupraHIIT in healthy older adults (Frykholm et al., 2024; Jakobsson et al., 2025; Simonsson et al., 2023). Thus, in our study we did not perform data-extrapolation used by Hedlund et al. (Hedlund et al., 2019) and Borg (Borg, 1982), but we applied the formula from Simonsson et al. to the highest achieved mBCST workload to calculate maximum MPO6 and subsequently the SupraHIIT intensity (Frykholm et al., 2024; Simonsson et al., 2023) (see template mBCST). In detail, SupraHIIT intensities were prescribed by multiplying the highest achieved BCST workload by 1.75 to estimate maximum mean power output over 6 seconds (MPO6), and subsequently this value is used to calculate the target power output corresponding to a certain percentage of maximum MPO6 (Frykholm et al., 2024; Jakobsson et al., 2025; Simonsson et al., 2023).

**Instructions:** Participants were instructed to increase their cadence during the last five seconds of the passive rest period to reach 80 to 90 RPM by the time the interval started. During each interval, participants maintained a constant cadence of 80 to 90 RPM, while the ergometer automatically adjusted the power output according to the set workload.

**Monitoring:** Blood pressure was obtained in the last minute of rest, every other passive rest period, at the end of the test-phase, and in the last minute of cool-down. RPE and rating of dyspnoea and leg fatigue (Borg CR10) were obtained in the last minute of rest, immediately after every incremental step during the test-phase, at the end of the test-phase, and in the last minute of cool-down. Inspiratory capacity manoeuvres were performed every minute during rest, at the end of every other incremental step, and in the last minute of cool-down (Guenette et al., 2013).

### Template mBCST

The modified Borg cycle strength test (mBCST) is performed to determine the power output somebody can maintain for 30 seconds corresponding to an RPE = 17. Next, the highest achieved workload can be used for determination of supramaximal intensities. For our purpose, the maximum MPO6 can be determined by multiplying the highest achieved workload during mBCST x 1.75 (Simonsson et al., 2023). The MPO6 is used to determine the target intensity for SupraHIIT. Starting intensity: 60% of MPO6. Gradual progression to 80% of MPO6.

**Example:** *mBCST achieved peak workload = 150 W.*

*MPO6 = mBCST achieved peak workload x 1.75 = 150 W x 1.75 = 263 W*

*Starting intensity = 60% MPO6 = 0.60 x 263W = 158 W*

*Gradual progression to 80% MPO6 = 0.80 x 263W = 210 W*

**Protocol mBCST**

1. Calibrate cycle ergometer

2. Make sure participant has followed preparatory instructions

3. Adjust seat and handlebars of cycle ergometer. Equip the participant with monitoring equipment (for example: heart rate sensor, pulse oximeter, blood pressure cuff) and explain scales (for example: Borg CR10 dyspnoea, leg fatigue) depending on need/indication.

4. Explain Borg RPE scale

5. Estimate a suitable workload step increment based on a previously obtained maximal aerobic power (MAP) during a maximal incremental cycle test or your judgement of the individuals’ physical capacity. A simplified version is provided below, while the used version in our study can be found at the end of the mBCST template.

| Wpeak | < 50W | 50 - 100W | 101 - 150 W | >150 |
| --- | --- | --- | --- | --- |
| mBCST step increment | 15W | 25W | 40W | 50W |

6. Perform a familiarization bout at the workload of the first step of the mBCST. Let the individual pedal up to 85 RPM (range 80 – 90 RPM).

7. Start the test (see table below) with at least three minutes of seated rest on the cycle ergometer. Ask Borg RPE at the end of the rest phase. Take additional measures if needed.

8. At the end of the rest phase provide the following instruction “*gradually* *ramp up to 85 RPM”* 5 to 10 seconds before the first interval will start. Let the individual cycle for 30 seconds at the target workload and RPM. Instruct the individual to “*keep legs still*” during the 30 seconds of recovery. Take Borg RPE rating immediately after the 30 second interval. Take additional measures if needed. We advise to obtain other rating scales after obtainment of the RPE rating.

9. At the end of the recovery phase provide the following instruction “*gradually* *ramp up to 85 RPM”* 5 to 10 seconds before the next interval will start. Let the individual cycle for 30 seconds at the target workload and RPM. Instruct the individual to “*keep legs still*” during the 30 seconds of recovery. Take Borg RPE rating immediately after the 30 second interval. Take additional measures if needed. We advise to obtain blood pressure every other recovery interval.

10. Repeat until Borg RPE 17 is rated immediately after an interval or until the cadence drops > 5 seconds under 75 RPM during an interval.

11. Perform at least 3 minutes of unloaded cool-down. Ask Borg RPE at the end of the cool-down phase. Take additional measures if needed.

**mBCST achieved workload**

- If the individual scored Borg RPE = 17, take the achieved workload during the last step of the mBCST
- If the individual stopped due to a drop in cadence > 5 seconds under 75 RPM:
  - If the individual cycled <10 seconds >75 RPM, the achieved workload of the previous mBCST step is taken
  - If the individual cycled ≥10 seconds >75 RPM, use the table below to add a certain watt to the achieved workload of the previous mBCST step. A simplified version is provided below, while the used version in our study can be found at the end of the mBCST template.

|  |  | **PROTOCOL** | | | |
| --- | --- | --- | --- | --- | --- |
| **Seconds** | **% of stage** | **15** | **25** | **40** | **50** |
| 11.00 | 0.37 | 6 | 9 | 15 | 18 |
| 12.00 | 0.40 | 6 | 10 | 16 | 20 |
| 13.00 | 0.43 | 7 | 11 | 17 | 22 |
| 14.00 | 0.47 | 7 | 12 | 19 | 23 |
| 15.00 | 0.50 | 8 | 13 | 20 | 25 |
| 16.00 | 0.53 | 8 | 13 | 21 | 27 |
| 17.00 | 0.57 | 9 | 14 | 23 | 28 |
| 18.00 | 0.60 | 9 | 15 | 24 | 30 |
| 19.00 | 0.63 | 10 | 16 | 25 | 32 |
| 20.00 | 0.67 | 10 | 17 | 27 | 33 |
| 21.00 | 0.70 | 11 | 18 | 28 | 35 |
| 22.00 | 0.73 | 11 | 18 | 29 | 37 |
| 23.00 | 0.77 | 12 | 19 | 31 | 38 |
| 24.00 | 0.80 | 12 | 20 | 32 | 40 |
| 25.00 | 0.83 | 13 | 21 | 33 | 42 |
| 26.00 | 0.87 | 13 | 22 | 35 | 43 |
| 27.00 | 0.90 | 14 | 23 | 36 | 45 |
| 28.00 | 0.93 | 14 | 23 | 37 | 47 |
| 29.00 | 0.97 | 15 | 24 | 39 | 48 |
| **ADD AMOUNT OF WATT TO PREVIOUS COMPLETED STAGE** | | | | | |

|  | **mBCST** | **Total time** | **Borg RPE (points)** | **Additional measure** | **Additional measure** | **Additional measure** |
| --- | --- | --- | --- | --- | --- | --- |
|  | Rest (0:00 – 3:00) | 0:00 – 3:00 |  |  |  |  |
| **1** | ______W (0:00 – 0:30) | 3:00 – 3:30 |  |  |  |  |
|  | Unloaded (0:30 – 1:00) | 3:30 – 4:00 |  |  |  |  |
| **2** | ______W (1:00 – 1:30) | 4:00 – 4:30 |  |  |  |  |
|  | Unloaded (1:30 – 2:00) | 4:30 – 5:00 |  |  |  |  |
| **3** | ______W (2:00 – 2:30) | 5:00 – 5:30 |  |  |  |  |
|  | Unloaded (2:30 – 3:00) | 5:30 – 6:00 |  |  |  |  |
| **4** | ___ W (3:00 – 3:30) | 6:00 – 6:30 |  |  |  |  |
|  | Unloaded (3:30 – 4:00) | 6:30 – 7:00 |  |  |  |  |
| **5** | ______W (4:00 – 4:30) | 7:00 – 7:30 |  |  |  |  |
|  | Unloaded (4:30 – 5:00) | 7:30 – 8:00 |  |  |  |  |
| **6** | ______W (5:00 – 5:30) | 8:00 – 8:30 |  |  |  |  |
|  | Unloaded (5:30 – 6:00) | 8:30 – 9:00 |  |  |  |  |
| **7** | ______W (6:00 – 6:30) | 9:00 – 9:30 |  |  |  |  |
|  | Unloaded (6:30 – 7:00) | 9:30 – 10:00 |  |  |  |  |
| **8** | ______W (7:00 – 7:30) | 10:00 – 10:30 |  |  |  |  |
|  | Unloaded (7:30 – 8:00) | 10:30 – 11:00 |  |  |  |  |
| **9** | ______W (8:00 – 8:30) | 11:00 – 11:30 |  |  |  |  |
|  | Unloaded (8:30 – 9:00) | 11:30 – 12:00 |  |  |  |  |
| **10** | ______W (9:00 – 9:30) | 12:00 –12:30 |  |  |  |  |
|  | Unloaded (9:30 – 10:00) | 12:30 – 13:00 |  |  |  |  |
|  | STOP (if incomplete step) |  |  |  |  |  |
|  | Cool-down (0:00 – 3:00) | 13:00 – 16.00 |  |  |  |  |

**mBCST results**

Last step (number):  complete  incomplete

🡺 If complete step 🡺 mBCST WR final step: W

🡺 If incomplete step 🡺 duration of last step: sec

🡺 duration of last step < 10 sec

🡺 mBCST WR of last complete step: W

🡺 duration of last step ≥ 10 sec

🡺 mBCST WR of last complete step + additional watt:

W

Reason of test termination when incomplete step (breathlessness, leg discomfort, both or other):

Remarks mBCST:

**Workload step increment calculator mBCST (study version)**


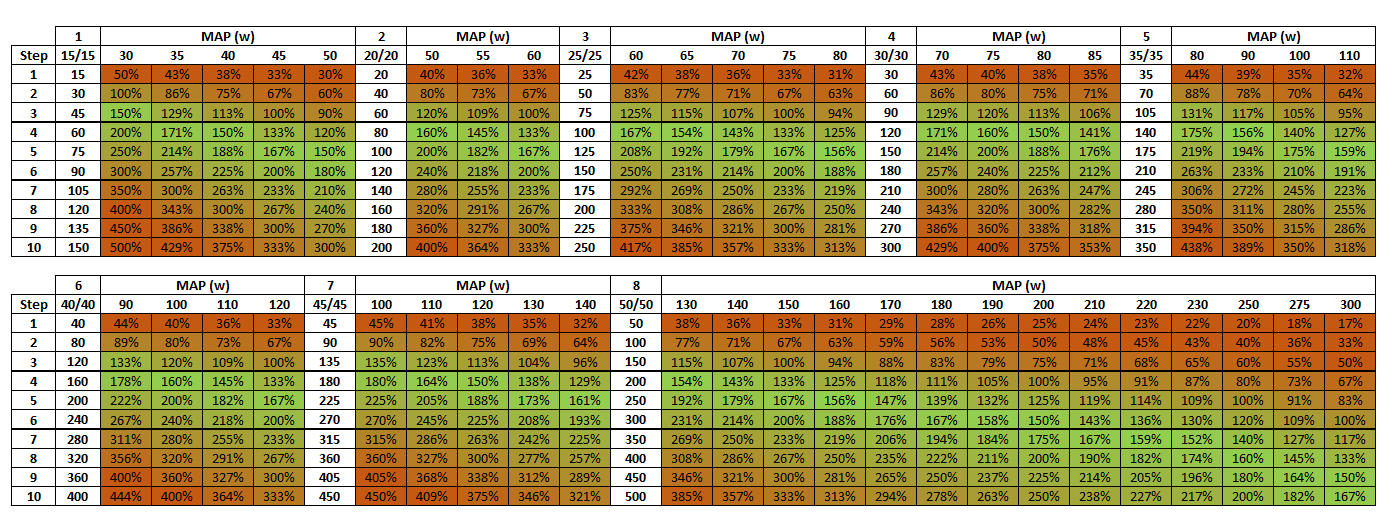


**mBCST achieved workload (incomplete step ≥ 10 seconds) (study version)**

|  |  |  | **PROTOCOL** | | | | | | |
| --- | --- | --- | --- | --- | --- | --- | --- | --- | --- |
| **Seconds** | **% of stage** | **15** | **20** | **25** | **30** | **35** | **40** | **45** | **50** |
| 11.00 | 0.37 | 6 | 7 | 9 | 11 | 13 | 15 | 17 | 18 |
| 12.00 | 0.40 | 6 | 8 | 10 | 12 | 14 | 16 | 18 | 20 |
| 13.00 | 0.43 | 7 | 9 | 11 | 13 | 15 | 17 | 20 | 22 |
| 14.00 | 0.47 | 7 | 9 | 12 | 14 | 16 | 19 | 21 | 23 |
| 15.00 | 0.50 | 8 | 10 | 13 | 15 | 18 | 20 | 23 | 25 |
| 16.00 | 0.53 | 8 | 11 | 13 | 16 | 19 | 21 | 24 | 27 |
| 17.00 | 0.57 | 9 | 11 | 14 | 17 | 20 | 23 | 26 | 28 |
| 18.00 | 0.60 | 9 | 12 | 15 | 18 | 21 | 24 | 27 | 30 |
| 19.00 | 0.63 | 10 | 13 | 16 | 19 | 22 | 25 | 29 | 32 |
| 20.00 | 0.67 | 10 | 13 | 17 | 20 | 23 | 27 | 30 | 33 |
| 21.00 | 0.70 | 11 | 14 | 18 | 21 | 25 | 28 | 32 | 35 |
| 22.00 | 0.73 | 11 | 15 | 18 | 22 | 26 | 29 | 33 | 37 |
| 23.00 | 0.77 | 12 | 15 | 19 | 23 | 27 | 31 | 35 | 38 |
| 24.00 | 0.80 | 12 | 16 | 20 | 24 | 28 | 32 | 36 | 40 |
| 25.00 | 0.83 | 13 | 17 | 21 | 25 | 29 | 33 | 38 | 42 |
| 26.00 | 0.87 | 13 | 17 | 22 | 26 | 30 | 35 | 39 | 43 |
| 27.00 | 0.90 | 14 | 18 | 23 | 27 | 32 | 36 | 41 | 45 |
| 28.00 | 0.93 | 14 | 19 | 23 | 28 | 33 | 37 | 42 | 47 |
| 29.00 | 0.97 | 15 | 19 | 24 | 29 | 34 | 39 | 44 | 48 |
|  |  |  | **ADD AMOUNT OF WATT TO PREVIOUS COMPLETED STAGE** | | | | | | |

### Outcome variables CPET and mBCST: data processing and presentation

**Table S1:** Data processing of outcome variables CPET and mBCST

| **Outcome variable** | **Data processing in Excel** |
| --- | --- |
| V̇O_2_, HR, V̇O_2_/HR, V̇E, RR, SpO_2_, V̇E/V̇O_2_, V̇E/V̇CO_2_, RER | 1. Outlier elimination (values outside 1.5 x the interquartile range below/above the first/third quartile) on raw breath-by-breath data  2. Smoothing of raw breath-by-breath data by 10 breaths moving average  3. isoMAP: Determination of the data point with the highest V̇O_2_ value during the isoMAP stage and its accompanying outcome variable values  4. End-mBCST and end-CPET: Determination of the data point with the highest V̇O_2_ value and its accompanying outcome variable values |
| V̇E/V̇CO_2_ nadir* | 1. Outlier elimination (values outside 1.5 x the interquartile range below/above the first/third quartile) on raw breath-by-breath data  2. Smoothing of raw breath-by-breath data by 10 breaths moving average  3. Determination of the data point with the lowest V̇E/V̇CO_2_ value |
| Δ_end-rest_ IC** | 1. Visual inspection of exercise flow-volume loops and evaluation of IC manoeuvres and number of stable breaths before every IC manoeuvre  2. Determination of resting IC: at least two acceptable IC manoeuvres with at least two stable breaths before IC manoeuvre, with a reproducibility criterion of CV <10%. The highest IC value is obtained.  3. Determination of end-exercise IC: acceptable IC manoeuvre with at least two stable breaths before IC manoeuvre.  4. Calculation of Δ_end-rest_ IC: end-exercise IC minus rest IC |
| TTE, MAP or workload, SBP, RPE, Borg D, Borg F | No data processing – value at isoMAP (mBCST) or end of exercise (mBCST and CPET) |

*Symbols*: * = V̇E/V̇CO_2_ nadir only retrieved from CPET; ** = Δ_end-rest_ IC was not calculated for CPET due to a large amount of invalid data (COPD: n = 13; healthy: n = 11). *Abbreviations:* V̇O_2_ = rate of oxygen consumption; HR = heart rate; V̇O_2_/HR = oxygen pulse; V̇E = ventilation; RR = respiratory rate; SpO_2_ = peripheral oxygen saturation; V̇E/V̇O_2_ = ventilatory equivalent oxygen; V̇E/V̇CO_2_ = ventilatory equivalent carbon dioxide; RER = respiratory exchange ratio; Δ_end-rest_ IC = delta end-exercise minus rest inspiratory capacity; TTE = time to exhaustion; MAP = maximal aerobic power; SBP = systolic blood pressure; RPE = rate of perceived exertion; Borg D = Borg dyspnoea; Borg F = Borg leg fatigue; IC = inspiratory capacity; CV = coefficient of variance.

## **Results**

**Table S2:** CPET

|  | ***CPET*** | |
| --- | --- | --- |
|  | **COPD**  **(n = 18)** | **Healthy older adults**  **(n=16)** |
| **Maximal effort achieved?** | | |
| **MAP %pred ≥ 100% (N[%])** | 4 [22] | 15 [94] |
| **V̇O_2_peak %pred ≥ 100% (N[%])** | 6 [33] | 11 [69] |
| **HRpeak %pred ≥ 100% (N[%])** | 2 [11] | 5 [31] |
| **V̇E/MVV ≥ 85% (N[%])^a^** | 15 [88] | 9 [56] |
| **Borg Dyspnea > ULN (N[%])^b^** | 14 [78] | 4 [25] |
| **RERpeak ≥ 1.05 (N[%])** | 8 [44] | 14 [88] |
| **At least one criteria achieved (N[%])** | 18 [100] | 16 [100] |
| **Reason for test termination^b^** | | |
| **Breathlessness (N[%])** | 7 [39] | 2 [13] |
| **Leg fatigue (N[%])** | 8 [44] | 9 [60] |
| **Breathlessness and leg fatigue (N[%])** | 1 [6] | 3 [20] |
| **Other reasons (N[%])^d^** | 2 [11] | 1 [7] |
| **Abnormal exercise performance?** | | |
| **V̇O_2_peak %pred < 85% (N[%])** | 7 [39] | 1 [6] |
| **MAP %pred < 85% (N[%])** | 9 [50] | 1 [6] |
| **Ventilatory limitation (N[%])^e,f^** | 9 [100] | 1 [100] |

Data is expressed as number [percentage]. *Symbols:* ^a^ = altered sample size (COPD: n = 17) due to MVV value not being available; ^b^ reference values of Ekström and colleagues (Ekstrom et al., 2024); ^c^= altered sample size (healthy: n = 15) due to reason of termination not obtained; ^d^= other reasons: breathlessness and leg discomfort combined with knee pain, breathlessness and leg discomfort combined with saddle pain, termination due to systolic blood pressure >250 mmHg; ^e^= only participants with an abnormal exercise performance included; ^f^ ventilatory limitation was assessed based on V̇E/MVV ≥ 85%, and drop in SPO_2_ ≥ 5% or Borg Dyspnea > ULN or V̇E/VCO2 > 34. *Abbreviations:* COPD = chronic obstructive pulmonary disease; MAP = maximal aerobic power; %pred = percentage predicted; V̇O_2_ = rate of oxygen consumption; HR = heart rate; V̇E = ventilation; MVV = maximal ventilatory ventilation obtained from MVV manoeuvre during lung function testing; ULN = upper limit of normal; RER = respiratory exchange ratio.

**Table S3:** CPET parameters

|  | ***CPET*** | | | |
| --- | --- | --- | --- | --- |
|  | **COPD**  **(n = 18)** | **Healthy older adults**  **(n = 16)** | **COPD vs healthy** | **P-value (2-sided)** |
| **Test duration and MAP** | | | | |
| **TTE (sec)** | 523 (162) | 642 (73) | -119 (-207 to -32) | **0.010** |
| **MAP (W)** | 111 (42) | 164 (38) | -52 (-80 to -25) | **< 0.001** |
| **MAP (%pred)** | 81 (23) | 116 (22) | -34 (-50 to -19) | **< 0.001** |
| **Values corresponding to V̇O_2_peak** | | | | |
| **V̇O_2_ (mL∙kg^-1^∙min^-1^)** | 21.1 (5.1) | 25.4 (3.8) | -4.3 (-7.5 to -1.1) | **0.010** |
| **V̇O_2_ (%pred)** | 93 (18) | 113 (19) | -19.4 (-32 to -6) | **0.004** |
| **HR (b∙min^-1^)** | 131 (19) | 148 (16) | -18 (-30 to -5) | **0.006** |
| **HR (%pred)** | 84 (13) | 95 (10) | -11 (-19 to -3) | **0.008** |
| **V̇O_2_/HR (mL/beat)** | 11.6 (3.1) | 13.0 (2.8) | -1.4 (-3.5 to 0.7) | 0.176 |
| **SBP (mmHg)** | 207 (27) | 215 (22) | -8 (-26 to 9) | 0.337 |
| **V̇E (L∙min^-1^)** | 64 (22) | 78 (22) | -14 (-29 to 2) | 0.081 |
| **V̇E (%MVV)^a^** | 99 (14) | 85 (20) | 14 (2 to 26) | **0.029** |
| **Respiratory rate (b∙min^-1^)** | 43 (10) | 38 (9) | 5 (-2 to 11) | 0.145 |
| **SpO_2_ (%)** | 97 (95 – 98) | 98 (95 – 99) | -1 (-2 to 1)* | 0.281 |
| **V̇E/V̇O_2_** | 38.8 (4.7) | 38.3 (7.0) | 0.5 (-3.7 to 4.6) | 0.823 |
| **V̇E/V̇CO_2_** | 36.7 (3.9) | 33.1 (5.3) | 3.6 (0.4 to 6.8) | **0.029** |
| **V̇E/V̇CO_2_ nadir** | 32.4 (30.5 – 34.7) | 28.5 (25.9 – 31.2) | 4.4 (2.0 to 6.9)* | **<0.001** |
| **RER** | 1.06 (0.09) | 1.16 (0.09) | -0.10 (-0.16 to -0.04) | **0.003** |
| **RPE (pt)** | 19 (17 – 20) | 17 (16 – 19) | 1 (0 to 3)* | **0.036** |
| **Borg dyspnoea (pt)** | 9 (8 – 10) | 7 (4 – 9) | 2 (0 to 3)* | **0.011** |
| **Borg leg fatigue (pt)** | 9 (7 – 10) | 7 (4 – 9) | 1 (0 to 4)* | 0.088 |

Between-group analysis was performed via Independent T-test or Welch’s test and data is expressed as mean (standard deviation) and mean difference (95% confidence interval), or performed via Mann-Whitney U test and data is expressed as median (quartile 1 – quartile 3) and Hodges-Lehman median difference (95% confidence interval). *Symbols*: ^a^ = altered sample size (COPD: n = 17) due to MVV value not being available; * = Hodges-Lehman median difference. *Abbreviations:* COPD = chronic obstructive pulmonary disease; TTE = time to exhaustion; MAP = maximal aerobic power; V̇O_2_ = rate of oxygen consumption; HR = heart rate; SBP = systolic blood pressure; V̇E = ventilation; MVV = maximal ventilatory ventilation obtained from MVV manoeuvre during lung function testing; SpO_2_ = peripheral oxygen saturation; V̇CO_2_ = rate of carbon dioxide production; RER = respiratory exchange ratio; RPE = rate of perceived exertion.

**Table S4:** Physiological response at end-exercise modified Borg cycle strength test and CPET

|  | **COPD (n = 16)** | | | | **Healthy older adults (n = 16)** | | | | **COPD vs healthy** | |
| --- | --- | --- | --- | --- | --- | --- | --- | --- | --- | --- |
|  | **End exercise**  **mBCST** | **End exercise**  **CPET** | **ΔmBCST-CPET**  **(95% CI)** | **P-value** | **End exercise**  **mBCST** | **End exercise**  **CPET** | **ΔmBCST-CPET**  **(95% CI)** | **P-value** | | **ΔmBCST-CPET**  **P-value** |
| **Workload (W)** | 175 (63) | 119 (37) | 56 (39 to 74) | **< 0.001** | 256 (55) | 164 (38) | 92 (79 to 105) | **<0.001** | | **0.002** |
| **V̇O_2_ (mL∙kg^-1^∙min^-1^)** | 20.9 (4.5) | 21.8 (5.0) | -0.9 (-1.8 to 0.1) | 0.068 | 25.7 (3.5) | 25.4 (3.8) | 0.3 (-1.2 to 1.7) | 0.709 | | 0.173 |
| **HR (b∙min^-1^)^a^** | 120 (104 – 130) | 135 (123 – 145) | -16 (-25 to -12)* | **< 0.001** | 135 (122 – 151) | 148 (138 – 156) | -14 (-17 to -10)* | **< 0.001** | | 0.520 |
| **V̇O_2_/HR (mL)^a^** | 13.0 (3.0) | 12.0 (3.2) | 1.0 (0.4 to 1.6) | **0.003** | 14.3 (2.9) | 13.0 (2.8) | 1.3 (0.3 to 2.3) | **0.016** | | 0.557 |
| **SBP (mmHg)** | 164 (27) | 212 (24) | -49 (-61 to -36) | **< 0.001** | 170 (29) | 215 (22) | -45 (-58 to -33) | **< 0.001** | | 0.688 |
| **V̇E (L∙min^-1^)** | 56 (47 – 68) | 60 (52 – 82) | -8 (-15 to -4)* | **0.002** | 69 (54 – 84) | 82 (58 – 98) | -11 (-16 to -4)* | **0.013** | | 0.445 |
| **V̇E (%MVV)^b^** | 82 (78 – 89) | 97 (85 – 111) | -13 (-20 to -7)* | **0.001** | 76 (62 – 87) | 87 (70 – 98) | -13 (-16 to -3)* | **0.015** | | 0.892 |
| **Respiratory rate (b∙min^-1^)** | 35 (31 – 43) | 41 (36 – 49) | -6 (-10 to -3)* | **0.002** | 36 (31 – 40) | 39 (30 – 46) | -4 (-6 to 0)* | **0.034** | | 0.086 |
| **ΔIC (L)^c^** | -0.50 (0.50) | - | - | - | -0.15 (0.44) | - | - | - | | - |
| **SpO_2_ (%)** | 95 (93 – 97) | 98 (95 – 98) | -2 (-4 to -1)* | **0.004** | 95 (94 – 96) | 98 (95 – 99) | -3 (-4 to -2)* | **0.011** | | 0.520 |
| **V̇E/V̇O_2_** | 35.7 (5.2) | 39.2 (4.8) | -3.4 (-5.6 to -1.3) | **< 0.001** | 33.4 (5.4) | 38.3 (7.0) | -4.9 (-7.2 to -2.6) | **< 0.001** | | 0.323 |
| **V̇E/V̇CO_2_** | 35.6 (5.1) | 36.5 (4.1) | -0.9 (-2.6 to 0.8) | 0.275 | 31.1 (4.6) | 33.1 (5.3) | -1.9 (-3.0 to -0.9) | **0.002** | | 0.276 |
| **RER** | 1.01 (0.08) | 1.07 (0.08) | -0.07 (-0.11 to -0.03) | **0.003** | 1.07 (0.05) | 1.16 (0.09) | -0.09 (-0.13 to -0.04) | **0.002** | | 0.573 |
| **RPE (pt)** | 17 (17 – 17) | 20 (18 – 20) | -2 (-3 to -1)* | **0.016** | 17 (15 – 17) | 17 (16 – 19) | -1 (-2 to -1)* | **0.003** | | 0.423 |
| **Borg dyspnoea (pt)** | 7 (7 – 9) | 9 (7 – 10) | -2 (-3 to 0)* | **0.029** | 6 (4 – 7) | 7 (4 – 9) | -1 (-2 to 0)* | **0.028** | | 0.669 |
| **Borg leg fatigue (pt)** | 7 (5 – 8) | 9 (7 – 10) | -2 (-3 to 0)* | **0.035** | 7 (4 – 7) | 7 (4 – 9) | -1 (-2 to 0)* | 0.070 | | 0.149 |

Within group data is analysed via Paired Sample T-test and expressed as mean (standard deviation) and mean difference (95% confidence interval) or analysed via Wilcoxon Rank test and expressed as median (quartile 1 – quartile 3) and Hodges-Lehman median difference (95% confidence interval). Between group ΔmBCST-CPET data is analysed via Independent T-test or Welch’s test, or Mann-Whitney U test. *Symbols^:^* ^a^ = altered sample size (COPD: n = 15) due to technical failure HR equipment; ^b^ = altered sample size (COPD: n = 15) due to MVV value not being available; ^c^ = altered sample size (COPD: n = 12; healthy: n = 10) due to invalid IC data for mBCST, and no CPET data available due to high number of invalid IC data; * = Hodges-Lehman median difference. *Abbreviations:* COPD = chronic obstructive pulmonary disease; V̇O_2_ = rate of oxygen consumption; HR = heart rate; SBP = systolic blood pressure; V̇E = ventilation; MVV = maximal voluntary ventilation obtained from MVV manoeuvre during lung function testing; ΔIC = delta inspiratory capacity (peak minus rest); SpO_2_ = peripheral oxygen saturation; V̇CO_2_ = volume of carbon dioxide production; RER = respiratory exchange ratio; RPE = rate of perceived exertion.

**Table S5:** Physiological response at iso-MAP modified Borg cycle strength test and end-exercise CPET

|  | **COPD (n = 16)** | | | | **Healthy older adults (n = 16)** | | | | **COPD vs healthy** | |
| --- | --- | --- | --- | --- | --- | --- | --- | --- | --- | --- |
|  | **Iso MAP**  **mBCST** | **End exercise**  **CPET** | **ΔmBCST-CPET**  **(95% CI)** | **P-value** | **Iso MAP**  **mBCST** | **End exercise**  **CPET** | **ΔmBCST-CPET**  **(95% CI)** | **P-value** | | **ΔmBCST-CPET**  **P-value** |
| **Workload (W)** | 117 (40) | 119 (37) | - | - | 163 (40) | 164 (38) | - | - | | - |
| **V̇O_2_ (mL∙kg^-1^∙min^-1^)** | 15.8 (3.0) | 21.8 (5.0) | -6.0 (-7.7 to -4.3) | **< 0.001** | 18.0 (3.8) | 25.4 (3.8) | -7.4 (-8.7 to -6.2) | **< 0.001** | | 0.167 |
| **HR (b∙min^-1^)^a^** | 106 (92 – 116) | 135 (123 – 145) | -32 (-38 to -25)* | **< 0.001** | 114 (104 – 122) | 148 (138 – 156) | -39 (-42 to -28)* | **< 0.001** | | 0.473 |
| **V̇O_2_/HR (mL)^a^** | 10.9 (2.8) | 12.0 (3.3) | -1.1 (-1.8 to -0.4) | **0.006** | 12.1 (2.8) | 13.0 (2.8) | -0.9 (-1.9 to 0.2) | 0.102 | | 0.707 |
| **V̇E (L∙min^-1^)** | 38 (31 – 46) | 60 (52 – 82) | -29 (-38 to -18)* | **< 0.001** | 33 (27 – 44) | 82 (58 – 98) | -43 (-50 to -34)* | **< 0.001** | | **0.023** |
| **V̇E (%MVV)^b^** | 55 (42 – 71) | 97 (85 – 111) | -41 (-52 to -30)* | **< 0.001** | 38 (34 – 44) | 87 (70 – 98) | -45 (-53 to -40)* | **< 0.001** | | 0.470 |
| **Respiratory rate (b∙min^-1^)** | 28 (24 – 35) | 41 (36 – 49) | -13 (-19 to -8)* | **< 0.001** | 26 (22 – 28) | 39 (30 – 46) | -12 (-15 to -9)* | **< 0.001** | | 0.780 |
| **SpO_2_ (%)** | 95 (93 – 96) | 98 (95 – 98) | -3 (-5 to -1)* | **0.008** | 95 (94 – 96) | 98 (95 – 99) | -3 (-4 to -2)* | **0.015** | | 0.956 |
| **V̇E/V̇O_2_** | 30.9 (6.3) | 39.2 (4.8) | -8.2 (-11.8 to -4.6) | **< 0.001** | 24.2 (4.0) | 38.3 (7.0) | -14.1 (-17.0 to -11.2) | **< 0.001** | | **0.011** |
| **V̇E/V̇CO_2_** | 36.5 (6.2) | 36.5 (4.1) | -0.0 (-2.5 to 2.4) | 0.970 | 28.7 (4.2) | 33.1 (5.3) | -4.3 (-5.9 to -2.8) | **< 0.001** | | **0.004** |
| **RER** | 0.84 (0.05) | 1.07 (0.08) | -0.23 (-0.28 to -0.18) | **< 0.001** | 0.84 (0.06) | 1.16 (0.09) | -0.31 (-0.36 to -0.27) | **< 0.001** | | **0.014** |
| **RPE (pt)** | 14 (13 – 15) | 20 (18 – 20) | -6 (-7 to -4)* | **< 0.001** | 13 (9 – 13) | 17 (16 – 19) | -6 (-7 to -5)* | **< 0.001** | | 0.423 |
| **Borg dyspnoea (pt)** | 4 (3 – 5) | 9 (7 – 10) | -5 (-6 to -4)* | **< 0.001** | 3 (2 – 3) | 7 (4 – 9) | -5 (-6 to -4)* | **< 0.001** | | 0.423 |
| **Borg leg fatigue (pt)** | 4 (3 – 5) | 9 (7 – 10) | -5 (-6 to -4)* | **< 0.001** | 2 (1 – 4) | 7 (4 – 9) | -4 (-5 to -4)* | **< 0.001** | | 0.361 |

Within group data is analysed via Paired Sample T-test and expressed as mean (standard deviation) and mean difference (95% confidence interval) or analysed via Wilcoxon Rank test and expressed as median (quartile 1 – quartile 3) and Hodges-Lehman median difference (95% confidence interval). Between group ΔmBCST-CPET data is analysed via Independent T-test or Welch’s test, or Mann-Whitney U test. IsoMAP mBCST was reached during step 3 or 4 of the mBCST protocol, except for one healthy older adult reaching isoMAP mBCST at step 5. Systolic blood pressure and inspiratory capacity data are not reported due to not being able to sample the data at isoMAP. *Symbols^:^* ^a^ = altered sample size (COPD: n = 15) due to technical failure HR equipment; ^b^ = altered sample size (COPD: n = 15) due to MVV value not being available; * = Hodges-Lehman median difference. *Abbreviations:* COPD = chronic obstructive pulmonary disease; V̇O_2_ = rate of oxygen consumption; HR = heart rate; V̇E = ventilation; MVV = maximal voluntary ventilation obtained from MVV manoeuvre during lung function testing; SpO_2_ = peripheral oxygen saturation; V̇CO_2_ = volume of carbon dioxide production; RER = respiratory exchange ratio; RPE = rate of perceived exertion

**Table S6:** mBCST parameters expressed as percentage of CPET

| ***modified Borg cycle strength test*** | | | | |
| --- | --- | --- | --- | --- |
|  | **COPD (n = 16)** | **Healthy older adults**  **(n = 16)** | **COPD vs Healthy** | |
|  | **Relative (%CPET)** | **Relative (%CPET)** | **Mean difference (95% CI)** | **P-value**  **(2-sided)** |
| **Workload peak (%CPET)** | 145 (125 – 168) | 154 (148 – 163) | -13 (-29 to 6)* | 0.239 |
| **V̇O_2_ peak (%CPET)** | 98 (90 – 103) | 100 (96 – 105) | -3 (-10 to 3)* | 0.287 |
| **HR peak (%CPET)^a^** | 87 (8) | 91 (5) | -4 (-8 to 1) | 0.128 |
| **V̇O_2_/HR peak (%CPET)^a^** | 105 (103 – 118) | 110 (100 – 117) | -2 (-10 to 9)* | 0.800 |
| **SBP peak (%CPET)** | 77 (10) | 79 (11) | -2 (-9 to 6) | 0.656 |
| **V̇E peak (%CPET)** | 89 (81 – 96) | 86 (79 – 94) | 2 (-7 to 10)* | 0.564 |
| **V̇E peak (%MVV) (%CPET)^b^** | 88 ( 81 – 95) | 86 (79 – 94) | 1 (-7 to 9)* | 0.711 |
| **Respiratory rate peak (%CPET)** | 88 (75 – 93) | 89 (83 – 98) | -6 (-13 to 3)* | 0.184 |
| **SpO_2_ peak (%CPET)** | 98 (96 – 100) | 97 (96 – 98) | 1 (-1 to 2)* | 0.572 |
| **V̇E/V̇O_2_ peak (%CPET)** | 91 (93 – 99) | 87 (79 – 96) | 4 (-4 to 11)* | 0.239 |
| **V̇E/V̇CO_2_ peak (%CPET)** | 98 (9) | 95 (6) | 3 (-2 to 8) | 0.268 |
| **RER peak (%CPET)** | 94 (7) | 93 (8) | 1 (-4 to 6) | 0.761 |
| **RPE peak (%CPET)** | 89 (85 – 100) | 89 (87 – 100) | 0 (-4 to 3)* | 0.590 |
| **Borg dyspnea peak (%CPET)** | 87 (70 – 100) | 89 (64 – 100) | 0 (-17 to 20)* | 0.897 |
| **Borg leg fatigue peak (%CPET)** | 75 (68 – 100) | 100 (78 – 100) | -13 (-30 to 0)* | 0.110 |

Between-group analysis was performed via Independent T-test or Welch’s test and data is expressed as mean (standard deviation) and mean difference (95% confidence interval), or performed via Mann-Whitney U test and data is expressed as median (quartile 1 – quartile 3) and Hodges-Lehman median difference (95% confidence interval). *Symbols*^: a^ = altered sample size (COPD: n = 15) due to technical failure HR equipment; ^b^ = altered sample size (COPD: n = 15) due to MVV value not being available; * = Hodges-Lehman median difference. *Abbreviations*: COPD = chronic obstructive pulmonary disease; V̇O_2_ = rate of oxygen consumption; HR = heart rate; SBP = systolic blood pressure; V̇E = ventilation; MVV = maximal ventilatory ventilation obtained from MVV manoeuvre during lung function testing; SpO_2_ = peripheral oxygen saturation; V̇CO_2_ = rate of carbon dioxide production; RER = respiratory exchange ratio; RPE = rate of perceived exertion.

**Table S7:** Association analysis between mBCST workload (%MAP) and participant characteristics in people with COPD

|  | **mBCST workload (%MAP)**  **Rho-value** | **P-value**  **(2-sided)** |
| --- | --- | --- |
| **Age (years)** | 0.138 | 0.611 |
| **Body weight (kg)** | 0.044 | 0.871 |
| **BMI (kg/m^2^)** | -0.262 | 0.327 |
| **Fat mass (%)** | -0.168 | 0.535 |
| **FFMI (kg/m^2^)** | -0.138 | 0.610 |
| **Packyears (N)** | -0.015 | 0.957 |
| **Total number of medications (N)** | -0.415 | 0.110 |
| **CAT score (points)** | -0.377 | 0.150 |
| **mMRC dyspnoea score (points)** | **-0.618** | **0.011** |
| **Step count (steps/day)** | 0.488 | 0.055 |
| **MVPA (min/day)** | **0.513** | **0.042** |
| **FEV_1_ (L)** | 0.282 | 0.289 |
| **FEV_1_ (%pred)** | 0.185 | 0.492 |
| **FVC (L)** | 0.244 | 0.362 |
| **FVC (%pred)** | 0.006 | 0.983 |
| **FEV_1_/FVC** | 0.153 | 0.572 |
| **IC (L)** | 0.085 | 0.753 |
| **DLCO (mmol/min/kPa)** | 0.300 | 0.259 |
| **DLCO (%pred)^a^** | **0.575** | **0.025** |
| **TLC (L)** | 0.153 | 0.572 |
| **TLC (%pred)^b^** | 0.038 | 0.901 |
| **FRC (L)** | 0.179 | 0.506 |
| **FRC (%pred)^b^** | -0.231 | 0.448 |
| **FRC/TLC** | -0.479 | 0.060 |
| **RV (L)** | 0.288 | 0.279 |
| **RV (%pred)^b^** | 0.209 | 0.494 |
| **RV/TLC** | -0.044 | 0.871 |
| **RV/TLC (%pred)^b^** | 0.148 | 0.629 |

Data is expressed Spearman rank correlation coefficient rho. Significant results are presented in bold and rho-values > 0.4 are coloured in grey. *Symbols:* ^a^ Altered sample size (COPD: n = 15) due to no Global Lung Initiative reference values for persons > 80 years old; ^b^ Altered sample size (COPD: n = 13) due to no Global Lung Initiative reference values for persons > 85 years old. *Abbreviations:* BMI = body mass index; FFMI = fat-free mass index; CAT = COPD assessment test; mMRC = modified medical research council; MVPA = moderate to vigorous physical activity; FEV_1_ = forced expiratory volume in one second; FVC = forced vital capacity; IC = inspiratory capacity; DLCO = diffusion capacity of the lung for carbon monoxide; TLC = total lung capacity; FRC = functional residual capacity; RV = residual volume.

**Table S8:** Association analysis between mBCST workload (%MAP) and CPET outcomes in people with COPD

|  | **mBCST workload (%MAP)**  **Rho-value** | **P-value**  **(2-sided)** |
| --- | --- | --- |
| **MAP (W)** | 0.353 | 0.180 |
| **MAP (%pred)** | 0.368 | 0.161 |
| **V̇O_2_ peak (mL∙kg^-1^∙min^-1^)** | 0.465 | 0.070 |
| **V̇O_2_ peak (%pred)** | 0.394 | 0.131 |
| **HR peak (b∙min^-1^)** | 0.376 | 0.151 |
| **HR peak (%pred)** | 0.376 | 0.151 |
| **V̇O_2_/HR peak (mL/beat)** | 0.188 | 0.485 |
| **SBP peak (mmHg)** | 0.232 | 0.388 |
| **V̇E peak (L∙min^-1^)** | 0.259 | 0.333 |
| **V̇E peak (%MVV)^a^** | -0.036 | 0.899 |
| **Respiratory rate peak (b∙min^-1^)** | 0.038 | 0.888 |
| **SpO_2_ peak (%)** | 0.379 | 0.148 |
| **V̇E/V̇O_2_ peak** | 0.085 | 0.753 |
| **V̇E/V̇CO_2_ peak** | -0.215 | 0.425 |
| **V̇E/V̇CO_2_ nadir** | **-0.588** | **0.017** |
| **RER peak** | 0.265 | 0.322 |
| **RPE peak (pt)** | -0.217 | 0.419 |
| **Borg dyspnoea peak (pt)** | **-0.566** | **0.022** |
| **Borg leg fatigue peak (pt)** | **-0.586** | **0.017** |
| **TTE (sec)** | 0.485 | 0.057 |

Data is expressed Spearman rank correlation coefficient rho. Significant results are presented in bold and rho-values > 0.4 are coloured in grey. *Symbols:* ^a^ = altered sample size (COPD: n = 15) due to MVV value not being available. *Abbreviations:* V̇O_2_ = rate of oxygen consumption; HR = heart rate; SBP = systolic blood pressure; V̇E = ventilation; MVV = maximal ventilatory ventilation obtained from MVV manoeuvre during lung function testing; SpO_2_ = peripheral oxygen saturation; V̇CO_2_ = rate of carbon dioxide production; RER = respiratory exchange ratio; RPE = rate of perceived exertion; TTE = time to exhaustion.

## **References**

Borg, G. (1982). Ratings of perceived exertion and heart rates during short-term cycle exercise and their use in a new cycling strength test. *International Journal of Sports Medicine*, *3*(3), 153-158. <https://doi.org/10.1055/s-2008-1026080>

Demeyer, H., Mohan, D., Burtin, C., Vaes, A. W., Heasley, M., Bowler, R. P., Casaburi, R., Cooper, C. B., Corriol-Rohou, S., Frei, A., Hamilton, A., Hopkinson, N. S., Karlsson, N., Man, W. D., Moy, M. L., Pitta, F., Polkey, M. I., Puhan, M., Rennard, S. I., . . . Troosters, T. (2021). Objectively Measured Physical Activity in Patients with COPD: Recommendations from an International Task Force on Physical Activity. *Chronic Obstr Pulm Dis*, *8*(4), 528-550. <https://doi.org/10.15326/jcopdf.2021.0213>

Ekstrom, M., Li, P. Z., Lewthwaite, H., Bourbeau, J., Tan, W. C., Schioler, L., Brotto, A., Stickland, M. K., Jensen, D., & Can, C. C. R. G. (2024). Normative Reference Equations for Breathlessness Intensity during Incremental Cardiopulmonary Cycle Exercise Testing. *Ann Am Thorac Soc*, *21*(1), 56-67. <https://doi.org/10.1513/AnnalsATS.202305-394OC>

Frykholm, E., Hedlund, M., Becker, C., Holmberg, H., Johansson, B., Klenk, J., Lindelöf, N., Lindemann, U., Simonsson, E., Boraxbekk, C. J., & Rosendahl, E. (2024). Effects of controlled supramaximal high-intensity interval training on muscle capacities and physical functions for older adults: analysis of secondary outcomes from the Umeå HIT study-a randomised controlled trial. *Age Ageing*, *53*(10). <https://doi.org/10.1093/ageing/afae226>

Graham, B. L., Brusasco, V., Burgos, F., Cooper, B. G., Jensen, R., Kendrick, A., MacIntyre, N. R., Thompson, B. R., & Wanger, J. (2017). 2017 ERS/ATS standards for single-breath carbon monoxide uptake in the lung. *Eur Respir J*, *49*(1). <https://doi.org/10.1183/13993003.00016-2016>

Guenette, J. A., Chin, R. C., Cory, J. M., Webb, K. A., & O'Donnell, D. E. (2013). Inspiratory Capacity during Exercise: Measurement, Analysis, and Interpretation. *Pulmonary Medicine*, *2013*, 956081. <https://doi.org/10.1155/2013/956081>

Hedlund, M., Lindelof, N., Johansson, B., Boraxbekk, C. J., & Rosendahl, E. (2019). Development and Feasibility of a Regulated, Supramaximal High-Intensity Training Program Adapted for Older Individuals. *Front Physiol*, *10*, 590. <https://doi.org/10.3389/fphys.2019.00590>

Jakobsson, J., De Brandt, J., Hedlund, M., Rullander, A.-C., Sandström, T., & Nyberg, A. (2025). Feasibility and acute physiological responses to supramaximal high-intensity interval-training in COPD: A randomised crossover trial. *ERJ Open Research*, 01321-02024. <https://doi.org/10.1183/23120541.01321-2024>

Jones, P. W., Harding, G., Berry, P., Wiklund, I., Chen, W.-H., & Kline Leidy, N. (2009). Development and first validation of the COPD Assessment Test. *European Respiratory Journal*, *34*(3), 648-654. <https://doi.org/10.1183/09031936.00102509>

Miller, M. R., Hankinson, J., Brusasco, V., Burgos, F., Casaburi, R., Coates, A., Crapo, R., Enright, P., van der Grinten, C. P., Gustafsson, P., Jensen, R., Johnson, D. C., MacIntyre, N., McKay, R., Navajas, D., Pedersen, O. F., Pellegrino, R., Viegi, G., Wanger, J., & Force, A. E. T. (2005). Standardisation of spirometry. *Eur Respir J*, *26*(2), 319-338. <https://doi.org/10.1183/09031936.05.00034805>

Radtke, T., Crook, S., Kaltsakas, G., Louvaris, Z., Berton, D., Urquhart, D. S., Kampouras, A., Rabinovich, R. A., Verges, S., Kontopidis, D., Boyd, J., Tonia, T., Langer, D., De Brandt, J., Goërtz, Y. M. J., Burtin, C., Spruit, M. A., Braeken, D. C. W., Dacha, S., . . . Hebestreit, H. (2019). ERS statement on standardisation of cardiopulmonary exercise testing in chronic lung diseases. *European Respiratory Review*, *28*(154), 180101. <https://doi.org/10.1183/16000617.0101-2018>

Simonsson, E., Levik Sandström, S., Hedlund, M., Holmberg, H., Johansson, B., Lindelöf, N., Boraxbekk, C. J., & Rosendahl, E. (2023). Effects of Controlled Supramaximal High-Intensity Interval Training on Cardiorespiratory Fitness and Global Cognitive Function in Older Adults: The Umeå HIT Study-A Randomized Controlled Trial. *J Gerontol A Biol Sci Med Sci*, *78*(9), 1581-1590. <https://doi.org/10.1093/gerona/glad070>

Stenton, C. (2008). The MRC breathlessness scale. *Occupational Medicine (Oxford, England)*, *58*(3), 226-227. <https://doi.org/10.1093/occmed/kqm162>

Wanger, J., Clausen, J. L., Coates, A., Pedersen, O. F., Brusasco, V., Burgos, F., Casaburi, R., Crapo, R., Enright, P., van der Grinten, C. P., Gustafsson, P., Hankinson, J., Jensen, R., Johnson, D., Macintyre, N., McKay, R., Miller, M. R., Navajas, D., Pellegrino, R., & Viegi, G. (2005). Standardisation of the measurement of lung volumes. *Eur Respir J*, *26*(3), 511-522. <https://doi.org/10.1183/09031936.05.00035005>
